# Exploring the impact of PDGFD in osteosarcoma metastasis through single-cell sequencing analysis

**DOI:** 10.1007/s13402-024-00949-3

**Published:** 2024-04-23

**Authors:** Yujing Huang, Dongyan Cao, Manxue Zhang, Yue Yang, Gengming Niu, Lina Tang, Zan Shen, Zhichang Zhang, Yueqing Bai, Daliu Min, Aina He

**Affiliations:** 1 Department of Oncology, Shanghai Sixth People’s Hospital, Shanghai Jiaotong University School of Medicine, Shanghai, China; 2 State Key Laboratory of Systems Medicine for Cancer, Shanghai Cancer Institute, Shanghai Jiaotong University School of Medicine, Shanghai, China; 3 Department of Biliary-Pancreatic Surgery, the Renji Hospital, Shanghai Jiaotong University School of Medicine, Shanghai, China; 4 Institute of Toxicology, School of Public Health, Lanzhou University, Lanzhou, Gansu Province China; 5 Shanghai OneTar Biomedicine, Shanghai, China; 6 Department of Orthopaedic, Shanghai Sixth People’s Hospital, Shanghai Jiaotong University School of Medicine, Shanghai, China; 7 Department of Pathology, Shanghai Sixth People’s Hospital, Shanghai Jiaotong University School of Medicine, Shanghai, China

**Keywords:** Osteosarcoma, Metastasis, scRNA-seq, PDGFD, Organoid

## Abstract

**Purpose:**

The overall survival rate for metastatic osteosarcoma hovers around 20%. Responses to second-line chemotherapy, targeted therapies, and immunotherapies have demonstrated limited efficacy in metastatic osteosarcoma. Our objective is to validate differentially expressed genes and signaling pathways between non-metastatic and metastatic osteosarcoma, employing single-cell RNA sequencing (scRNA-seq) and additional functional investigations. We aim to enhance comprehension of metastatic mechanisms and potentially unveil a therapeutic target.

**Methods:**

scRNA-seq was performed on two primary osteosarcoma lesions (1 non-metastatic and 1 metastatic). Seurat package facilitated dimensionality reduction and cluster identification. Copy number variation (CNV) was predicted using InferCNV. CellChat characterized ligand-receptor-based intercellular communication networks. Differentially expressed genes underwent GO function enrichment analysis and GSEA. Validation was achieved through the GSE152048 dataset, which identified PDGFD-PDGFRB as a common ligand-receptor pair with significant contribution. Immunohistochemistry assessed PDGFD and PDGFRB expression, while multicolor immunofluorescence and flow cytometry provided insight into spatial relationships and the tumor immune microenvironment. Kaplan-Meier survival analysis compared metastasis-free survival and overall survival between high and low levels of PDGFD and PDGFRB. Manipulation of PDGFD expression in primary osteosarcoma cells examined invasion abilities and related markers.

**Results:**

Ten clusters encompassing osteoblasts, osteoclasts, osteocytes, fibroblasts, pericytes, endothelial cells, myeloid cells, T cells, B cells, and proliferating cells were identified. Osteoblasts, osteoclasts, and osteocytes exhibited heightened CNV levels. Ligand-receptor-based communication networks exposed significant fibroblast crosstalk with other cell types, and the PDGF signaling pathway was activated in non-metastatic osteosarcoma primary lesion. These results were corroborated by the GSE152048 dataset, confirming the prominence of PDGFD-PDGFRB as a common ligand-receptor pair. Immunohistochemistry demonstrated considerably greater PDGFD expression in non-metastatic osteosarcoma tissues and organoids, correlating with extended metastasis-free and overall survival. PDGFRB expression showed no significant variation between non-metastatic and metastatic osteosarcoma, nor strong correlations with survival times. Multicolor immunofluorescence suggested co-localization of PDGFD with PDGFRB. Flow cytometry unveiled a highly immunosuppressive microenvironment in metastatic osteosarcoma. Manipulating PDGFD expression demonstrated altered invasive abilities and marker expressions in primary osteosarcoma cells from both non-metastatic and metastatic lesions.

**Conclusions:**

scRNA-seq illuminated the activation of the PDGF signaling pathway in primary lesion of non-metastatic osteosarcoma. PDGFD displayed an inhibitory effect on osteosarcoma metastasis, likely through the suppression of the EMT signaling pathway.

**Supplementary Information:**

The online version contains supplementary material available at 10.1007/s13402-024-00949-3.

## Introduction

Osteosarcoma is recognized as the predominant primary malignant bone tumor in the adolescent population, with an incidence rate of approximately 8.7 per million individuals [1]. This malignancy exhibits a high level of severity, characterized by rapid disease progression and an unfavorable prognosis, significantly impacting the overall well-being of patients and their families. The implementation of a multidisciplinary strategy, involving neoadjuvant chemotherapy, surgical intervention, and adjuvant chemotherapy, has contributed to an enhanced 5-year survival rate of approximately 70% for osteosarcoma [2, 3]. Patients with metastatic disease upon presentation have a significantly poorer prognosis compared to those with localized osteosarcoma, despite receiving the same treatment protocol [4, 5]. The overall survival rate for metastatic osteosarcoma is approximately 20% [6]. Limited efficacy has been observed in responses to second-line chemotherapy [7–11], targeted therapies [12–14], and immunotherapies [15–17] in the context of metastatic osteosarcoma. Consequently, there is an urgent need to elucidate the molecular mechanism underlying the metastasis of osteosarcoma and identify more efficacious therapeutic targets.

At the time of diagnosis, approximately 20% of patients exhibit metastatic disease, with a particular predilection for lung metastases, which serves as a crucial prognostic factor associated with an unfavorable outcome [18]. The metastatic lesion’s biological characteristics, including cell cycle, differentiation, karyotype, metabolism, and microenvironment, are significantly influenced by inherent genetic heterogeneity, alterations in molecular profile, and dynamic immunogenic features, leading to marked distinctions from the primary tumor [19–22]. Nevertheless, there remains a paucity of knowledge regarding the disparities between primary lesions with or without metastasis.

In recent years, there has been significant advancement in single-cell sequencing technology [23]. This technology is of particular significance in addressing the challenges associated with limited accessibility and biological heterogeneity of biological materials, as compared to traditional sequencing methods. By utilizing this technology, researchers are able to investigate the onset and progression of osteosarcoma at the individual cell level, encompassing aspects such as tumor metastasis, tumor evolution, carcinogenesis, and circulating tumor cells. Notably, single-cell RNA sequencing (scRNA-seq) has emerged as a promising tool for exploring intratumor heterogeneity in various cancers and the cellular cross-talk with the tumor microenvironment (TME) [24, 25]. This study utilized scRNA-seq and functional assays to elucidate the impact of various factors, including cell type composition, developmental stage, and metabolic alterations in the tumor immune microenvironment, on the initiation and progression of tumors [26]. The findings of this investigation shed light on differentially expressed genes and intercellular communication within primary tumor samples obtained from osteosarcoma patients, with and without metastasis, thereby offering substantial evidence to facilitate future osteosarcoma research.

## Patients and methods

### Human osteosarcoma samples

This study included two patients as the discovery cohort: one with histologically confirmed non-metastatic osteosarcoma and one with metastatic osteosarcoma. The validation cohort comprised 24 newly diagnosed cases between 2021 and 2023. The clinicopathological features of these patients were collected (Supplementary Table 1). Prior to sample collection, the study received approval from the Ethics Committee of Shanghai Sixth People’s Hospital Affiliated to Shanghai Jiaotong University School of Medicine (No.2018-072), and written informed consent was obtained from all patients or their guardians.

### Cell lines and cell culture

Human osteosarcoma cell lines (U2OS and Saos-2), rat osteosarcoma cell line UMR-106, and mouse osteosarcoma cell line K7M2 were purchased from the Cell Bank of Chinese Academy of Sciences. Human osteosarcoma cell line 143B was obtained from the American Type Culture Collection (ATCC) Cell Bank. Saos-2 was grown in McCoy’s 5a Medium supplemented with 1% penicillin/streptomycin (P/S) and 15% fetal bovine serum (FBS). U2OS was cultured in McCoy’s 5a Medium plus 1% P/S and 10% FBS. 143B, K7M2 and UMR-106 were maintained in Dulbecco’s modified Eagles medium (DMEM), 1% P/S and 10% FBS. All cell lines were grown at 37 °C with a 5% CO_2_ cell culture incubator. Cell culture reagents were purchased from Gibco, including FBS, McCoy’s 5a Medium, DMEM, and P/S.

### scRNA-seq and Library preparation

Fresh tumor tissue from primary osteosarcoma was immersed in a 10 cm dish filled with GEXSCOPETM tissue preservation solution (Singleron, China) and subsequently chilled on ice for processing. The samples were subjected to three washes with Hanks’ Balanced Salt Solution (HBSS; Gibco, USA), followed by fragmentation into 1–2 mm tissue fragments. Surgical forceps were employed to eliminate connective tissue and evident necrotic tissue. Tissue fragments were digested using 2 mL of GEXSCOPETM tissue dissociation solution (Singleron, China) at a temperature of 37 °C with agitation for a duration of 15 min. The resulting samples were then filtered through a 40 μm sterile filter and centrifuged at a speed of 800 × g for a period of 5 minutes. The supernatant was discarded, and the cell pellet was resuspended in 1 mL of phosphate-buffered saline (PBS; Gibco, USA). To remove erythrocytes, 2 mL of GEXSCOPETM erythrocyte lysis buffer (Singleron, China) was promptly added. The cell suspension was subjected to incubation at a temperature of 25 °C for a duration of 10 min, followed by centrifugation at a force of 500 × g for a period of 5 minutes. The resulting pellet was then resuspended in phosphate-buffered saline (PBS). Subsequently, the samples were stained with trypan blue (Gibco, USA) and the viability of the cells was evaluated using phase contrast light microscopy (Olympus, Japan). For the loading of single-cell suspensions (2 × 10^5^ cells/mL) onto the microwell chip, PBS (Gibco, USA) was employed in conjunction with the Singleron Matrix® Single Cell Processing System. The Barcoding Beads are retrieved from the microwell chip, and subsequently subjected to reverse transcription of the mRNA that has been captured by the Barcoding Beads, resulting in the generation of complementary DNA (cDNA). The cDNA is then amplified through polymerase chain reaction (PCR). Following amplification, the cDNA is fragmented and joined with sequencing adapters. The construction of scRNA-seq libraries adhered to the GEXSCOPE® Single Cell RNA Library Kits (Singleron, China) protocol. Each individual library was diluted to a concentration of 4 nM, pooled together, and subjected to sequencing on an Illumina novaseq 6000 platform, utilizing 150 bp paired end reads.

### scRNA-seq data processing

The fastq files of the raw data and feature matrices were obtained using CeleScope (v1.7.1). Subsequently, the data underwent processing using the R package Seurat (version 4.0.4). Filtering was performed to exclude low quality cells, defined as those with minimum expression cells greater than 3, gene numbers less than 200 or greater than 4000, and mitochondrial genes exceeding 15%. Following normalization of gene expression measurements, batch effects were eliminated using Harmony. The dimensionality reduction and cluster identification process were facilitated by the utilization of Seurat. Cell type annotation was conducted by relying on the known marker genes. The Copy number variation (CNV) levels of cell subtypes, namely osteocyte, osteoblast, and osteoclast, were calculated using InferCNV, with a reference set consisting of endothelial cells and macrophages. The intercellular communication networks based on ligand-receptor interactions were characterized using CellChat. To identify the enriched GO terms of biological processes, the differentially expressed genes underwent Gene Ontology (GO) function enrichment analysis. Gene set enrichment analysis (GSEA)was performed to complete GO enrichment analysis in all groups.

### Validation in GEO datasets

To ensure the reliability of our findings, we obtained a dataset of scRNA-seq (GSE152048[99]) from the GEO database (https://www.ncbi.nlm.nih.gov/geo/). A validation set comprising five samples (BC2, BC3, BC5, BC21, and BC22) was included. We employed standardized Seurat procedures for dimensionality reduction, cluster identification, and cell annotation, which align with our own single-cell transcriptome sequencing data.

### Osteosarcoma organoid culture

Patient-derived organoids are cultured with the organoid culture protocol and media produced by OneTar Biomedicine. In the context of organoid cultures, 4-mm-diameter pieces of tissues obtained from primary osteosarcomas were subjected to processing and subsequently washed with ice-cold PBS. The tumor pieces were then dissociated into individual cells using Tumor Tissue Digestion Solution (OneTar, China) under gentle shaking conditions at 37 °C for a duration of 40 min. Following cell counting, the cells were resuspended in Organoid Culture Matrigel (OneTar, China) and plated as Matrigel domes in 24-well tissue culture plates. The organoid cultures were maintained at 37 °C with 5% CO_2_, with Organoid Culture Media (OneTar, China) overlaying the Matrigel dome. Weekly monitoring was conducted to observe the growth of initiated organoids.

### Immunohistochemical analyses

Paraffin sections from primary osteosarcoma underwent deparaffinization, rehydration, and antigen retrieval. The sections were immersed in a 3% hydrogen peroxide solution for 5 min to block endogenous peroxidase activity. Subsequently, the sections were blocked with a 10% BSA solution in PBS for 60 min and incubated overnight at 4 °C with primary antibodies against PDGFD (Proteintech, 14075-1-AP, 1:100) and PDGFRB (Affinity, AF6133, 1:100). Following this, HRP-labeled polymer-conjugated secondary antibody was applied to the sections, and the samples were incubated at room temperature for 60 min. The samples were subsequently subjected to three rounds of rinsing in PBS, with each rinse lasting for a duration of 3 min. The immune complexes were then visualized through exposure to the DAB chromogenic agent for approximately 5 min. To facilitate visualization, the nuclei were counterstained with hematoxylin. A total of three random immunostaining images were captured from each specimen.

### Immunofluorescence staining

The sections from primary osteosarcoma were stained following the manufacturer’s guidelines of multiplex fluorescence immunohistochemical staining kit (RecordBio, RC0086-34RM). Sections were blocked with PBS containing 3% goat serum before incubation with antibodies. The primary antibodies involved in experiment include PDGFD (Proteintech, 14075-1-AP, 1:100), PDGFRB (Affinity, AF6133, 1:100), COL1a1 (Cell Signaling Technology/CST, 72,026 S, 1:200), CD4 (Abways, CY5392, 1:100), CD8 (Proteintech, 66868-1-Ig, 1:400), CD86 (Abways, CY5238, 1:100), and CD206 (Proteintech, 18704-1-AP, 1:400). The nuclei were stained with DAPI before sealing. A fluorescence secondary antibody and a quenching agent were added following removal of the primary antibodies. All sections were scanned by a fluorescent scanning camera (Nikon DS-U3).

### Survival analysis

The patients were divided into high-level and low-level groups based on the expression of PDGFD and PDGFRB. The overall survival (OS) was determined by calculating the time from the date of diagnosis to either the date of death from any cause or the date of the last follow-up. The metastasis-free survival (MFS) was calculated by measuring the time from the date of diagnosis to the occurrence of metastasis. The Kaplan-Meier method was employed to analyze both OS and MFS, and a log rank test was used to compare the obtained results.

### Multi-color flow cytometry analysis

Multi-color flow cytometry was employed to evaluate the proportions of different immune cell population. We stained osteosarcoma cells from primary lesions and matched peripheral blood mononuclear cells (PBMC) with a panel of antibodies, including CD45 (Biolegend, 1:100), CD3 (Biolegend, 1:100), CD4 (Biolegend, 1:100), CD8 (Biolegend, 1:100), CD56 (Biolegend, 1:100), CD11b (Biolegend, 1:100), CD68 (Biolegend, 1:100), CD86 (Biolegend, 1:100), CD206 (Biolegend, 1:100), and CD14 (Biolegend, 1:100). After that, flow cytometry (Attune NxT) was used to assess the cells. The data were analyzed using FlowJo v10.8.1.

### Primary osteosarcoma cell culture and transfection

Tissues derived from primary osteosarcoma were sectioned into 4-mm-diameter fragments and rinsed with ice-cold phosphate-buffered saline (PBS). The tumor fragments were then dissociated into individual cells using Tumor Tissue Digestion Solution (OneTar) for a duration of 40 min at 37 °C with gentle agitation. Subsequently, the cells were seeded onto culture flasks containing RPMI-1640 medium (Invitrogen) supplemented with 10% fetal bovine serum (Invitrogen), 100 µg/mL penicillin, and 0.1 mg/mL streptomycin (Sigma), and maintained in a humidified atmosphere of 5% CO_2_ at 37 °C. Upon reaching 80–90% confluence, the cells were subcultured into fresh flasks following trypsinization with 0.25% trypsin (Gibco).

The PDGFD shRNA plasmids and control shRNA plasmids, were purchased from Genechem Biotechnology. The primary osteosarcoma cells were transfected with PDGFD shRNA plasmids to generate shPDGFD group or control shRNA plasmids to generate shControl group according to the manufacturer’s instructions, when the cells had achieved 80% confluence. The PDGFD overexpressing cells were constructed as PDGFD OE group and Control group following the same procedure. shRNA sequences are listed as follows: sh-PDGFD, 5′-CCCAGGAATTACTCGGTCAAT-3′; sh-negative control, 5′-TTCTCCGAACGTGTCACGT-3′.

### Transwell assay

After a 3-day period of transfection, the cells were digested and collected, followed by resuspension in serum-free RPMI-1640 (Invitrogen). Subsequently, 1 × 10^6^ cells were seeded into the upper chamber (Corning, 3495) of a transwell system, while the lower chamber was supplemented with 500 µL of RPMI-1640 (Invitrogen) containing 10% fetal bovine serum (Invitrogen). The 24-well plates were then placed in a humidified atmosphere of 5% CO_2_ at 37 °C. Following a 48-hour incubation period, the cells were fixed with 4% paraformaldehyde for 1 h, washed thrice with PBS, stained with Gimsa dye for 30 minutes at room temperature, and washed thrice with PBS.

### RNA isolation and quantitative real-time PCR (RT-qPCR)

The extraction of total RNA from transfected cells was carried out using TRIzol reagent (Invitrogen) following the manufacturer’s protocols. Subsequently, the extracted RNA was reverse transcribed to cDNA using the M-MLV Reverse Transcriptase (Vazyme). The RT-qPCR analysis was conducted using the PrimeScript RT Master Mix Kit (Takara). The housekeeping gene GAPDH was utilized as an internal control. The resulting data were obtained as Ct values, which were then employed to calculate ∆Ct values. The relative gene expressions, normalized to GAPDH mRNA levels, were determined using the 2^−ΔΔCt^ method. The primer sequences for all RT-qPCR reactions are given in Supplementary Table 2.

### Statistical analysis

The statistical analysis was performed using GraphPad Prism 9.0 software. An unpaired Student t-test was used to compare numerical data, and data are presented as the mean ± SD. Fisher’s exact test was applied to analyze the categorical data. P value < 0.05 is considered statistically significant.

## Results

**Fig. 1:**
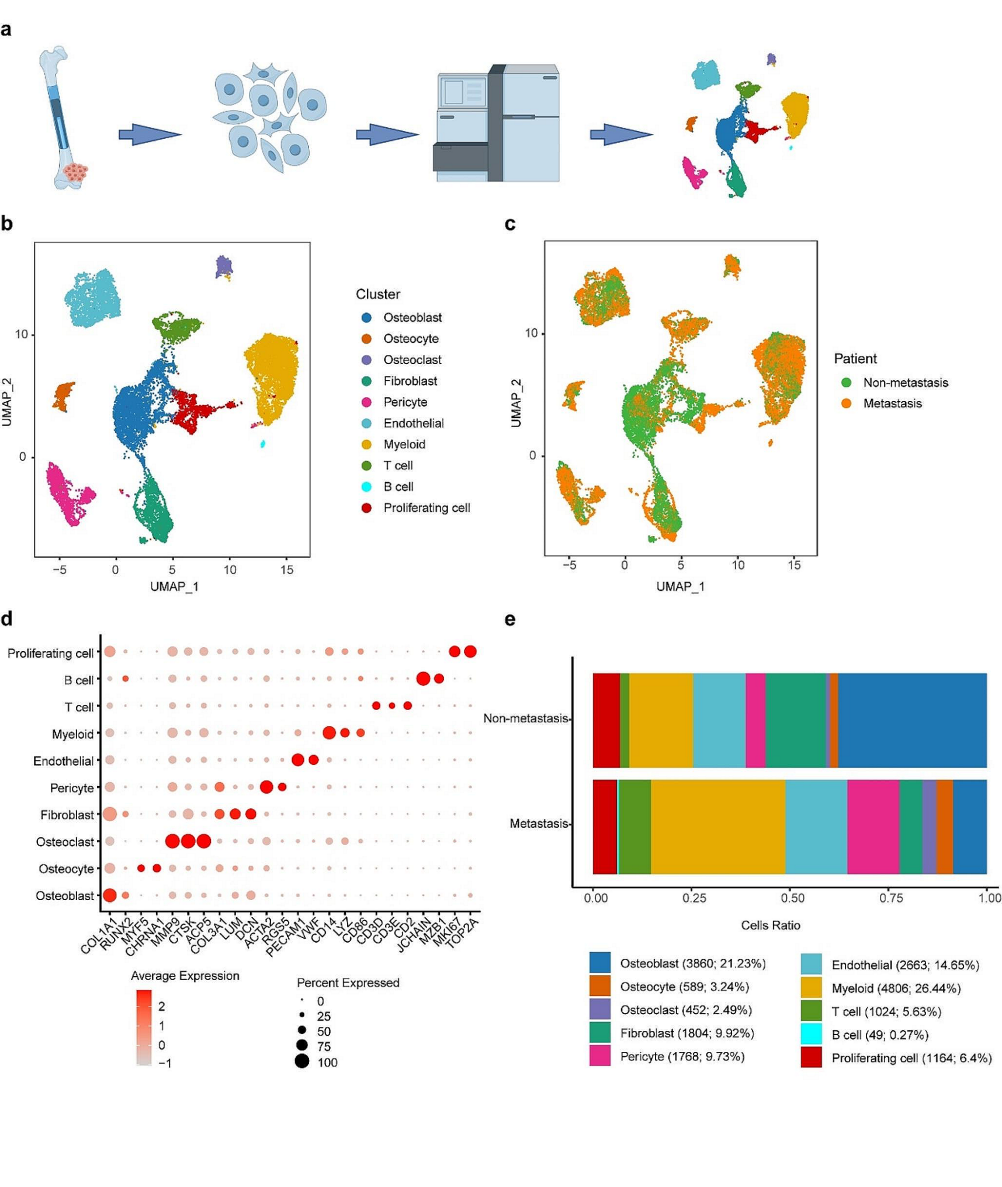
Cell profiling in osteosarcoma assessed by scRNA-seq. **a** Flow chart for the collection and processing of primary lesions from patients with osteosarcoma for scRNA-seq. **b** UMAP plot displayed ten identified 10 cell types. **c** UMAP plot based on samples. **d** Dot plots showed the signature gene expressions across the 10 cell types. **e** The relative proportions of cell types in each sample

### The landscape of the osteosarcoma microenvironment by single-cell-based profiling

In order to investigate the cellular composition of osteosarcoma, we procured a primary lesion of non-metastatic osteosarcoma and a primary lesion of metastatic osteosarcoma, which were promptly digested to yield a single-cell suspension. Following a rigorous quality filtering process, we successfully acquired single-cell transcriptomes from a total of 18,179 cells. Utilizing scRNA-seq analysis, we conducted an unbiased clustering of the cells in parallel based on their gene expression profiles and canonical markers, employing Seurat. As a result, we identified 10 distinct cell types within the osteosarcoma samples. In particular, they were as follows: osteoblasts highly expressing *COL1A1* and *RUNX2*; osteocytes with high expression of *MYF5* and *CHRNA1*; osteoclasts characterized with *MMP9*, *CTSK*, and *ACP5*; fibroblasts highly expressing *COL3A1*, *LUM*, and *DCN*; pericytes with high expression of *ACTA2* and *RGS5*; endothelia cells characterized by *PECAM1* and *VWF*; myeloid cells specifically expressing *CD14*, *LYZ*, and *CD86*; T cells highly expressing *CD3D*, *CD3E*, and *CD2*; B cells with high expression of *JCHAIN* and *MZB1*; and proliferating cells characterized by *MKI67* and *TOP2A*. We then analysis the myeloid cell clusters, and identified macrophages with high expression of *CD14*, *C1QA*, *C1QB*, and *C1QC*; CD14^+^ monocytes highly expressing *FCN1*, *S100A8*, and *S100A9*; and dendritic cells characterized with *CD1C*, *FCER1A*, and *CLEC10A* (Fig. 1, Supplementary Table 3).

Re-clustering of the myeloid cell subset identified the presence of macrophages, CD14^+^ monocytes, and dendritic cells (Supplementary Fig. 1, Supplementary Table 4). Analysis of the gene CNV found CNV was present in osteoblasts, osteoclasts and osteoblasts (Supplementary Fig. 2).

### Cell communication network analysis

**Fig. 2:**
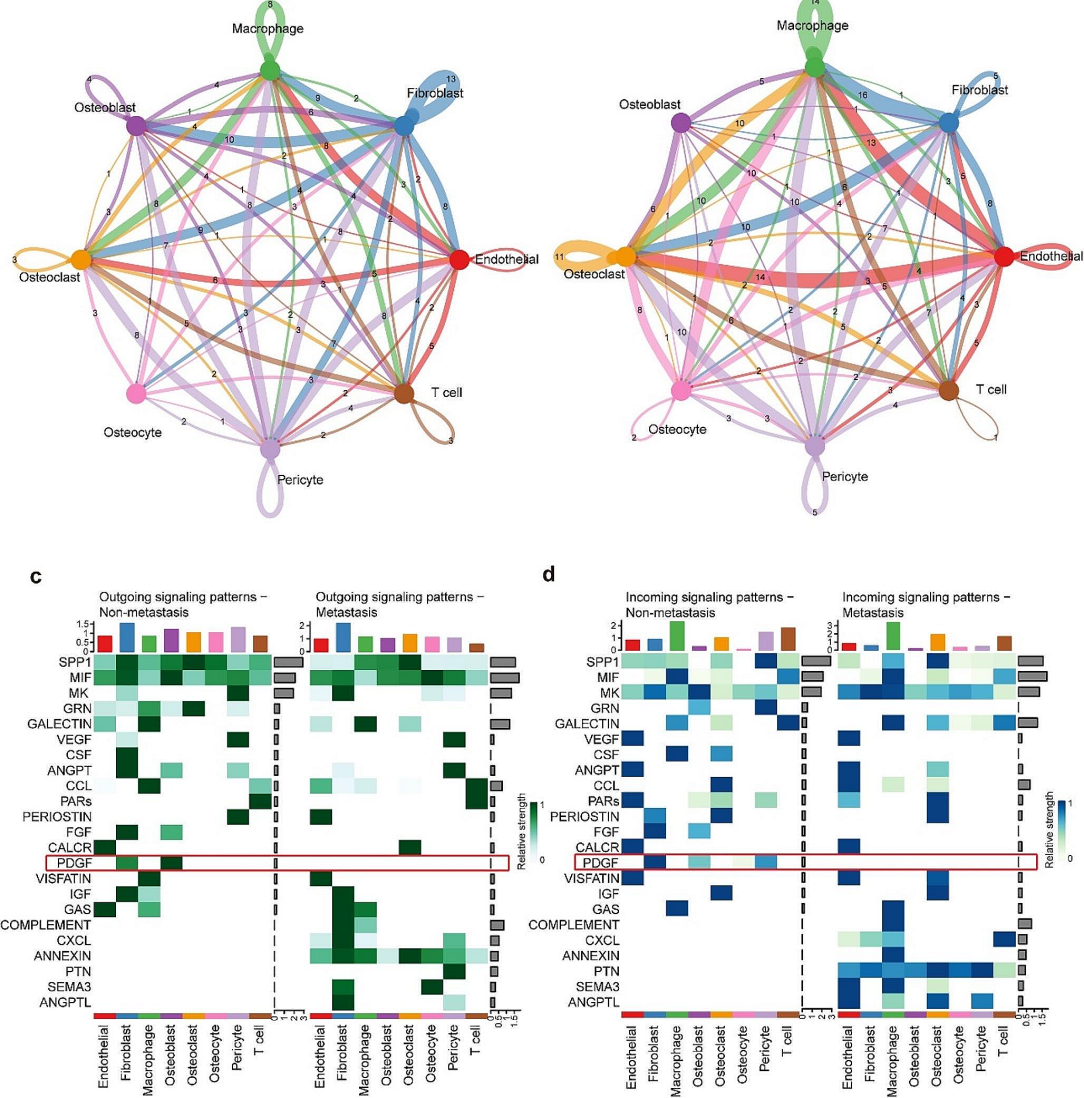
Cell communication network analysis. **a** Connectome networks in non-metastatic osteosarcoma. Dots indicate cell types. The thickness of line is proportional to the number of interactions between two cell types. The loops indicate autocrine circuits. **b** Connectome networks in metastatic osteosarcoma. **c** Heatmap of the outgoing signaling patterns. **d** Heatmap of the incoming signaling patterns

The findings from the analysis conducted using CellChat software indicate that fibroblasts exhibit a higher degree of intercellular communication with other cells, particularly fibroblasts and osteoblasts, in non-metastasis osteosarcoma sample compared to metastatic osteosarcoma (Fig. 2a, b; Supplementary Tables 5, 6). Additionally, a more detailed examination of the outgoing and incoming signaling within different cellular subpopulations in these pathways reveals the presence of the PDGF signaling pathway in the sample derived from non-metastatic osteosarcoma primary lesion. Signals were transmitted from fibroblasts and osteoblasts to fibroblasts, osteoblasts and pericytes. Conversely, no significant outgoing or incoming PDGF signaling was observed in metastatic osteosarcoma primary lesion (Fig. 2c, d).

### PDGFD-PDGFRB is the specific ligand-receptor pair in non-metastatic osteosarcoma

**Fig. 3:**
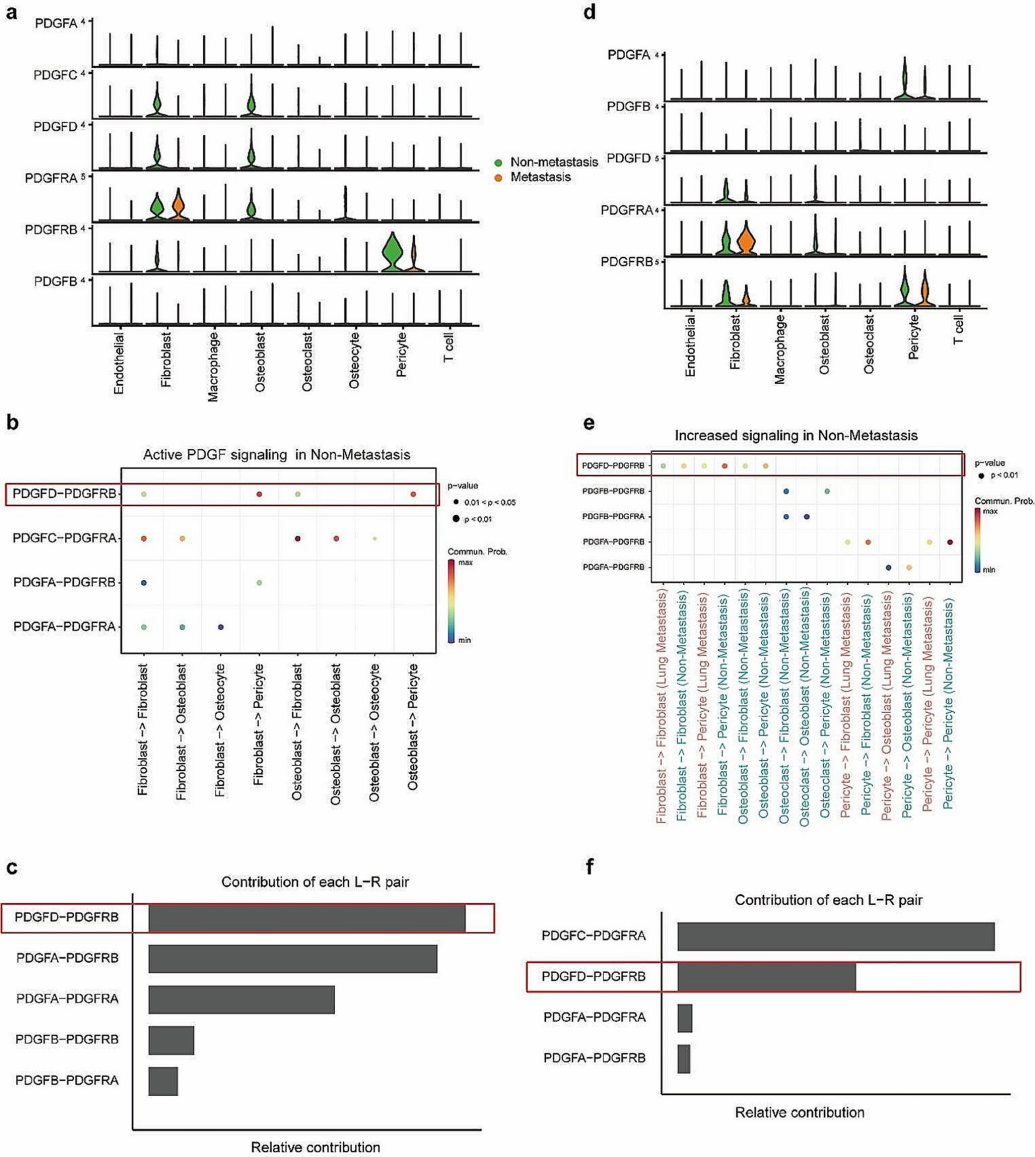
The identification of the crucial PDGF-PDGFR pair. **a** Violin plots showed the expression of different subtypes of PDGF-PDGFR pair in discovery cohort dataset. **b** Dot plot depicted different subtypes of PDGF-PDGFR pair in discovery cohort dataset. The dot color and size represent the calculated communication probability and p-values. **c** Relative contribution of each PDGF-PDGFR pair in discovery cohort dataset. **d** Violin plots showed the expression of different subtypes of PDGF-PDGFR pair in GSE152048 dataset. **e** Dot plot depicted different subtypes of PDGF-PDGFR pair in GSE152048 dataset. **f** Relative contribution of each PDGF-PDGFR pair in GSE152048 dataset

To identify the ligands and receptors of the PDGF pathway, we generated violin maps of the expression of different subtypes of PDGF-PDGFR pair in each cellular subpopulation (Fig. 3a) and compared the communication probabilities mediated by different subtypes of PDGF-PDGFR pair between cellular subpopulations (Fig. 3b). Signals delivered from fibroblasts, possibly via the PDGFD-PDGFRB, PDGFC-PDGFRA, PDGFA-PDGFRB, and PDGFA-PDGFRA to fibroblasts; via PDGFA-PDGFRA and PDGFC-PDGFRA to osteoblasts; via PDGFA-PDGFRA to osteocytes; and via PDGFD-PDGFRB and PDGFA-PDGFRB to pericytes. Fibroblasts received signals, and their biological behavior can be regulated by PDGFD-PDGFRB and PDGFC-PDGFRA. Osteoblasts transmitted signals to fibroblasts via PDGFD-PDGFRB and PDGFC-PDGFRA, to osteoblasts and osteocytes via PDGFC-PDGFRA, and to pericytes via PDGFD-PDGFRB. Osteoblasts received signals from others via PDGFA-PDGFRA and PDGFC-PDGFRA (Fig. 3b).

Cell clustering of the sequencing data of the five samples included from the GSE152048 dataset identified 10 cell subpopulations, namely osteoblasts, osteoclasts, fibroblasts, pericytes, endothelial cells, myeloid cells, T cells, mast cells, proliferating cells, and undefinable cell populations. Re-clustering of the myeloid cell subset identified the presence of macrophages, CD14^+^ monocytes, and dendritic cells (Supplementary Fig. 3). We then generated violin maps of the expression of different subtypes of PDGFD-PDGFRB pair in each cellular subpopulation (Fig. 3c) and compared communication probabilities mediated by different subtypes of PDGFD-PDGFRB pair between cellular subpopulations (Fig. 3d). Comparison of the contribution of each ligand-receptor pair of the PDGF signaling pathway in the non-metastatic osteosarcoma samples from the GSE152048 dataset and our sequencing samples indicated that the PDGFD-PDGFRB was the common ligand-receptor pair with a relatively large contribution (Fig. 3e, f).

### GO analysis and GSEA analysis

In fibroblasts, enriched biological processes among upregulated genes were multicellular organism metabolic process, regulation of protein activation cascade, angiogenesis, extracellular matrix disassembly, negative regulation of cytokine production, positive regulation of epithelial cell proliferation, positive regulation of cell migration, negative regulation of immune system process, and negative regulation of apoptotic signaling pathway. Enriched biological processes among downregulated genes were osteoblasts differentiation, blood vessel morphogenesis, skeletal system morphogenesis, regulation of DNA metabolic process, bone development, mesenchymal cell differentiation, and extracellular matrix organization. In osteoblasts, upregulated genes were enriched in co-translational protein targeting to membrane, translational initiation, multi-organism metabolic process, nuclear-transcribed mRNA catabolic process, and negative regulation of intrinsic apoptotic signaling pathway, whereas osteoblast differentiation, extracellular structure organization, extracellular matrix organization, regulation of ossification, skeletal system morphogenesis, blood vessel morphogenesis, negative regulation of cell growth, regulation of epithelial to mesenchymal transition, cell junction organization, and regulation of cytoskeleton organization showed enrichment for the downregulated genes (Supplementary Fig. 4).

Additional GSEA showed, downregulation of establishment or maintenance of cell polarity and osteoblasts differentiation, and upregulation of positive regulation of locomotion and regulation of vasculature development were present in fibroblasts of metastatic samples, compared with non-metastatic samples (Supplementary Fig. 5). In osteoblasts of metastatic samples, cell adhesion, extracellular matrix assembly, and ossification were downregulated, and negative regulation of immune response was upregulated (Supplementary Fig. 6).

### PDGFD was highly expressed in non-metastatic osteosarcoma

**Fig. 4:**
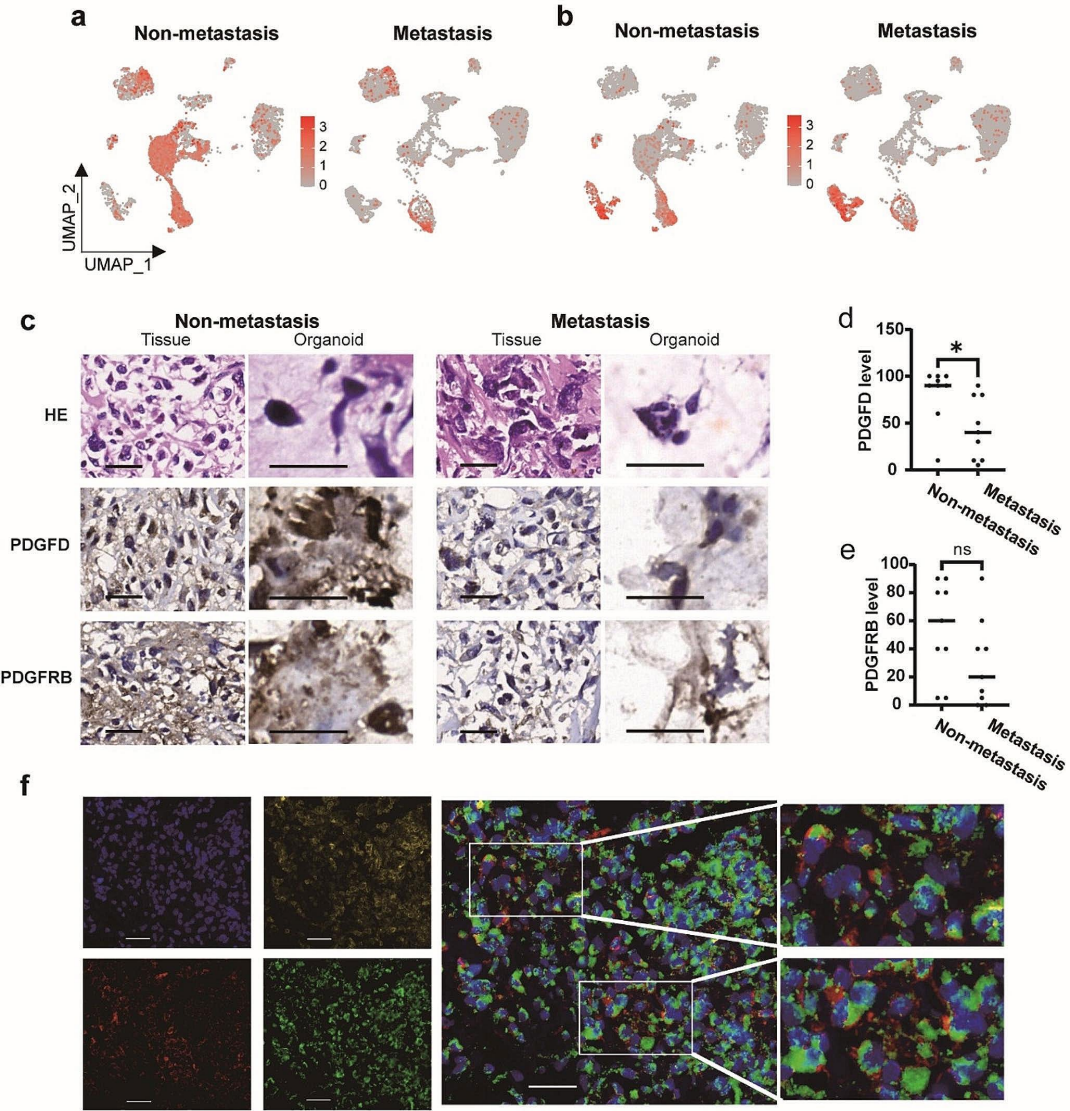
The expression of PDGFD and PDGFRB in osteosarcoma. **a** UMAP plot displayed PDGFD highly expressed in the non-metastatic osteosarcoma. **b** UMAP plot displayed similar expression of PDGFRB in the non-metastatic and metastatic osteosarcoma. **c** Immunohistochemical staining for PDGFD and PDGFRB in samples and organoids. Black scale bars: 30 μm. **d** PDGFD positivity was significantly higher in non-metastatic osteosarcoma primary lesions than metastatic primary lesions. **e** The differing positive rate of PDGFRB between non-metastatic osteosarcoma primary lesions and metastatic primary lesions was not statistically significant. * *p* < 0.05. ns, not significant, *p* > 0.05. **f** Multi-color immunofluorescence analysis revealed that PDGFD and PDGFRB were co-localized. Color code: Blue = DAPI, Red = COL1a1, Green = PDGFD, Yellow = PDGFRB. White scale bars: 40 μm.

scRNA-seq data analysis revealed that PDGFD expression levels were significantly higher in non-metastatic osteosarcoma, compared with metastatic samples (Fig. 4a). However, the expression of PDGFRB didn’t show significantly difference in non-metastatic and metastatic samples (Fig. 4b). And the mRNA levels of PDGFD and PDGFRB in human osteosarcoma cells lines (143B, U2OS, and Saos-2), rat osteosarcoma cell line UMR-106, and mouse osteosarcoma cell line K7M2 were shown in Supplementary Fig. 7. IHC was used to detect PDGFD and PDGFRB expression in non-metastatic and metastatic osteosarcoma primary lesions. and found that PDGFD was mainly expressed in osteosarcoma cells, while the PDGFRB expression was observed in both osteosarcoma cells and stromal cells (Fig. 4c). PDGFD positivity was significantly higher in non-metastatic osteosarcoma primary lesions than metastatic primary lesions (81.67% ± 21.58% vs. 43.89% ± 33.15%, *p* = 0.0214, Fig. 4d). The differing positive rate of PDGFRB between non-metastatic osteosarcoma primary lesions and metastatic primary lesions was not statistically significant (54.44% ± 33.86% vs. 29.44% ± 30.87%, *p* = 0.1212, Fig. 4e). Similar results were obtained in organoids cultured from surgical specimens of non-metastatic osteosarcoma primary lesions and metastatic osteosarcoma primary lesions (Fig. 4c). Multi-color immunofluorescence staining of PDGFD, PDGFRB, and COL1a1 was performed, and co-localization of PDGFD and PDGFRB is indicated, suggesting that PDGFD is binding to PDGFRB (Fig. 4f).

### Overexpression of PDGFD was associated with improved survival

18 patients were divided into high-level and low-level groups based on the expression of PDGFD and PDGFRB. The associations between PDGFD and PDGFRB and clinical features were assessed using the Fisher’s exact test. The low and high PDGFD level groups did not differ significantly with any of the clinical characteristics, with fewer metastasis in high level group (*p* = 0.050). Similarly, there were no significant differences in clinical characteristics by group in PDGFRB (Table 1).

**Table 1:** Comparison of patient characteristics between groups

Clinical characteristics	PDGFD high	PDGFD low	*p*	PDGFRB high	PDGFRB low	*p*
Gender			1.000			0.367
Male	5	4		2	6	
Female	6	3		5	5	
Age(year)			0.596			0.326
≥ 18	7	6		4	9	
< 18	4	1		3	2	
Location			-			-
Limbs	11	7		7	11	
Others	0	0		0	0	
Pathology			-			-
Conventional	11	7		7	11	
Others	0	0		0	0	
Neoadjuvant chemotherapy			1.000			0.389
Yes	10	7		6	11	
No	1	0		1	0	
Diameter(cm)			0.145			0.367
> 8	3	5		2	6	
≤ 8	8	2		5	5	
Necrosis rate			0.485			0.110
≥ 90%	2	1		1	0	
< 90%	8	6		5	11	
Recurrence			1.000			1.000
Yes	2	2		2	2	
No	9	5		5	9	
Metastasis			0.050			0.335
Yes	3	6		2	7	
No	8	1		5	4	

**Fig. 5:**
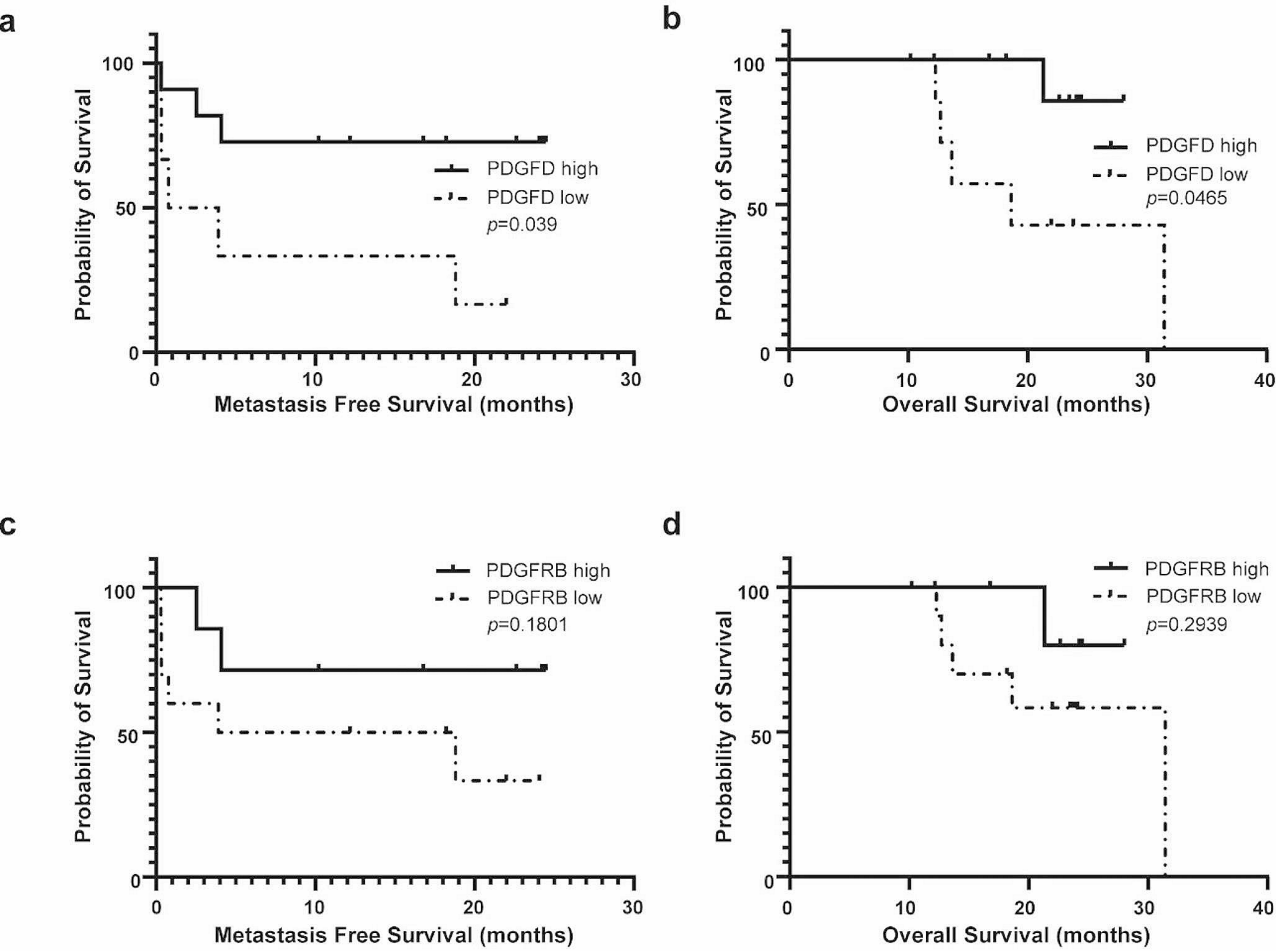
Kaplan-Meier survival curves of metastasis-free survival and overall survival. **a** Metastasis-free survival was significantly longer in the PDGFD-high group than in the PDGFD-low group. **b** Overall survival was significantly longer in the PDGFD-high group than in the PDGFD-low group. **c** Metastasis-free survival was not significantly different between PDGFRB-high group and the PDGFRB-low group. **d** Overall survival was not significantly different between PDGFRB-high group and the PDGFRB-low group.

According to the Kaplan-Meier survival analysis, the median MFS of low level of PDGFD was 2.335 months. Median MFS was not reached for high level of PDGFD. Patients with high level of PDGFD had significantly longer MFS than low-level group (*p* = 0.039, Fig. 5a). The median OS of high-level group is significantly longer compared with the low-level group (18.6 months vs. not reached, *p* = 0.0465, Fig. 5b). The median MFS in the low level PDGFRB group was 11.35 months and was not reached in the high-level group. The median MFS between high-level group and low-level group was not statistically significant (*p* = 0.1801, Fig. 5c). Similar results were found in media OS (31.43 months vs. not reached, *p* = 0.2939, Fig. 5d).

### The immunosuppressive microenvironment in osteosarcoma

**Fig. 6:**
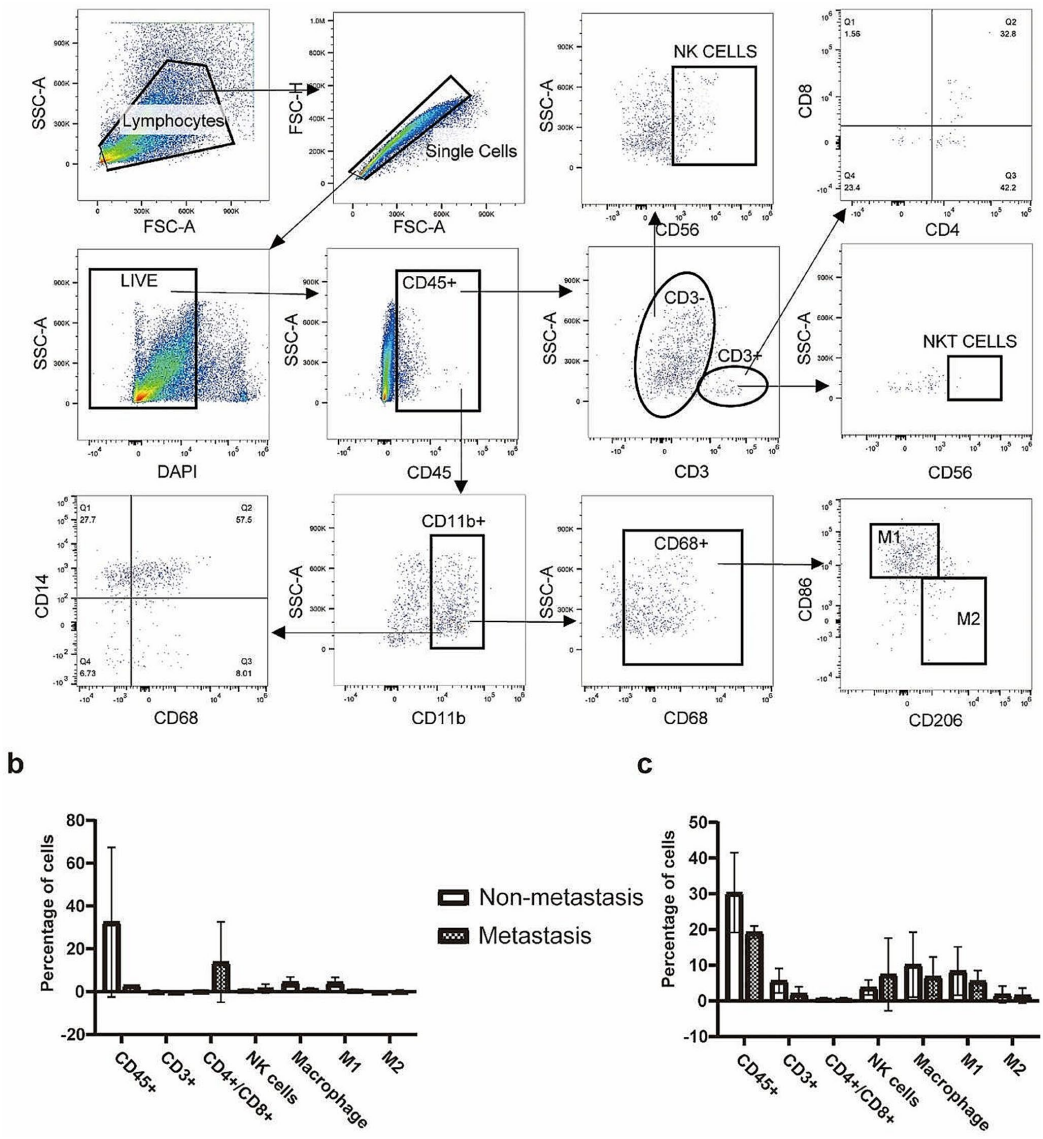
The highly immunosuppressive microenvironment in metastatic osteosarcoma. **a** The gating strategy of multicolor flow cytometry. **b** Bar chart of the percentage of immune cell subsets in primary lesions of osteosarcoma. **c** Bar chart of the percentage of immune cell subsets in peripheral blood mononuclear cells of osteosarcoma

Multi-color flow cytometry was performed to assess different immune cell subsets, and the gating strategies were shown in Fig. 6a. The percentages of CD45^+^ cells (32.46% vs. 3.096%), macrophage (4.536% vs. 1.312%), and M1 macrophage (4.359% vs. 0.999%) were higher in samples from non-metastatic osteosarcoma primary lesions than those in metastatic osteosarcoma primary lesions (Fig. 6b). Similarly, PBMC samples from non-metastatic osteosarcoma showed higher percentages of CD45^+^ cells (30.34% vs. 19.24%), CD3^+^ cells (5.662% vs. 2.057%), macrophage (10.19% vs. 6.849%), and M1 macrophage (8.378% vs. 5.620%) than those from metastatic osteosarcoma. These findings indicated a highly immunosuppressive microenvironment in metastatic osteosarcoma (Fig. 6c).

**Fig. 7:**
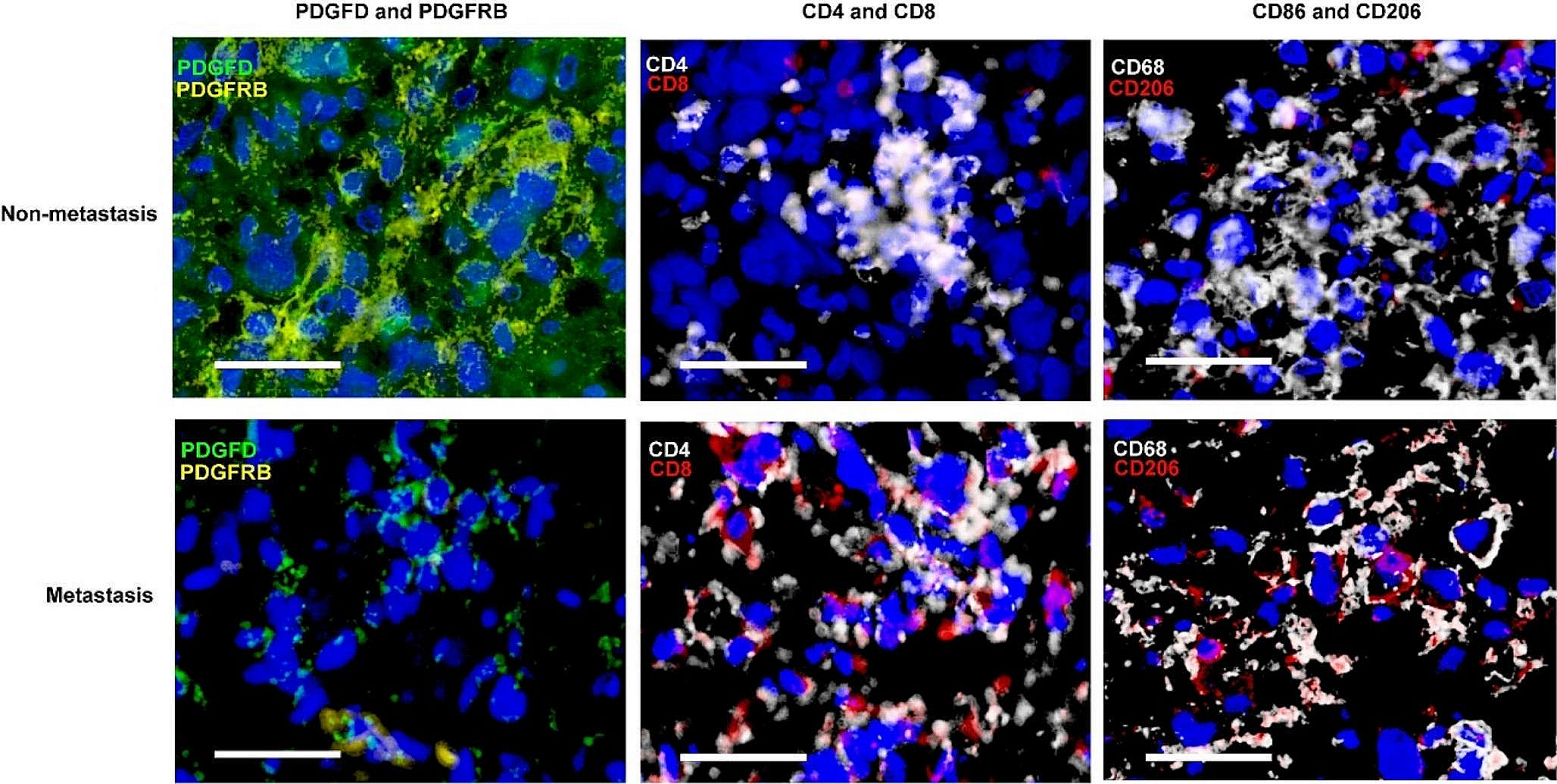
The relationship between PDGFD-PDGFRB and tumor immune microenvironment in osteosarcoma. Color code: Blue = DAPI, Green = PDGFD, Yellow = PDGFRB, White = CD4 or CD86, Red = CD8 or CD206. White scale bars: 40 μm

Studies have shown that the tumor microenvironment exerts momentous roles in osteosarcoma development and metastasis [27]. The higher level of infiltration of immune cells in the microenvironment predicted the better the prognosis in osteosarcoma [28, 29]. Han and Wolf-Dennen et al. found that the expression of cytokines, chemokines, and cell markers associated with M2 macrophages increased in lung metastasis lesion of osteosarcoma [30, 31]. Similarly, Dhupkar et al. found that the transition from the M2 macrophage phenotype to the M1 macrophage phenotype can reduce the occurrence of lung metastasis in osteosarcoma [32]. Furthermore, a multifactorial regression analysis of 376 advanced osteosarcoma patients found that the CD4^+^/CD8^+^ is an independent prognostic factor for osteosarcoma [33].PDGFD played a regulatory role in gastric cancer and pancreatic cancer by interfering with the tumor microenvironment [34, 35]. Moreover, as one of the immune-related genes, it involved in the predictive models of in bladder cancer [36, 37], Renal clear cell carcinoma [38], and osteosarcoma [39]. However, the relationship between PDGFD-PDGFRB and tumor immune microenvironment has not previously been reported. Here, we explored its relevance in immune microenvironment by staining with fluorescent-tagged antibodies to CD4, CD8, CD86, and CD206, and the results were shown in Fig. 7.

### Manipulating PDGFD could alter invasive abilities and marker expressions

**Fig. 8:**
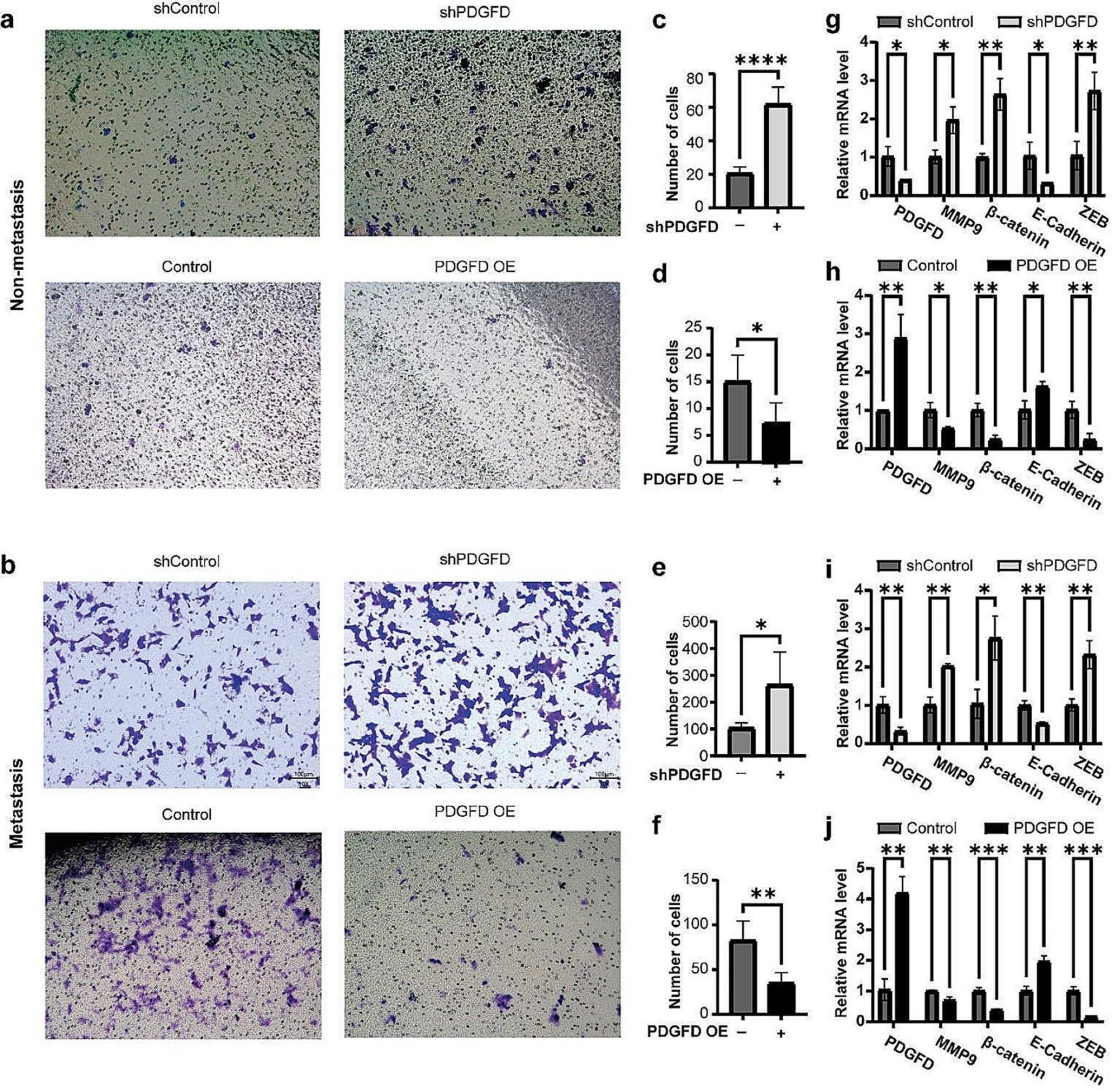
PDGFD inhibit the metastasis ability of osteosarcoma. **a** Invasion assays for primary osteosarcoma cells derived from non-metastatic osteosarcoma with PDGFD overexpression or knockdown. **b** Invasion assays for primary osteosarcoma cells derived from metastatic osteosarcoma with PDGFD overexpression or knockdown. **c-d** bar graph showed the number of invade cells in primary osteosarcoma cells derived from non-metastatic osteosarcoma with PDGFD overexpression or knockdown. **e-f** bar graph showed the number of invade cells in primary osteosarcoma cells derived from metastatic osteosarcoma with PDGFD overexpression or knockdown. **g-h** Bar chart of fold change of GAPDH-normalized mRNA levels of indicated genes in primary osteosarcoma cells derived from non-metastatic osteosarcoma with PDGFD overexpression or knockdown. **i-j** Bar chart of fold change of GAPDH-normalized mRNA levels of indicated genes in primary osteosarcoma cells derived from metastatic osteosarcoma with PDGFD overexpression or knockdown. * *p* < 0.05, ** *p* < 0.01, *** *p* < 0.001, **** *p* < 0.0001

A transwell assay was utilized to investigate PDGFD’s effects on metastatic capacity in primary osteosarcoma cells in vitro. In primary cells derived from non-metastatic osteosarcoma, the number of invaded cells per field of shPDGFD group was higher than that of shControl group (61.8 ± 9.13 vs. 6 ± 3.56, *p* < 0.0001, Fig. 8a, c), and PDGFD OE group had less invaded cells per field than control group (7.4 ± 3.26 vs. 15 ± 4.43, *p* = 0.0245, Fig. 8a, d). Similar results were obtained in primary cells derived from metastatic osteosarcoma. The number of invaded cells per field of shPDGFD group was higher than that of shControl group (264 ± 110.02 vs. 103.6 ± 17.50, *p* = 0.021, Fig. 8b, e), and PDGFD OE group had less invaded cells per field than control group (35 ± 10.71 vs. 82.8 ± 19.58, *p* = 0.003, Fig. 8b, f). These results suggested PDGFD knockdown significantly promoted metastasis, while PDGFD overexpression inhibited metastasis.

the downregulation of PDGFD, regardless of non-metastatic osteosarcoma or metastatic osteosarcoma, led to a significant upregulation of matrix metallopeptidase 9(MMP9), β-catenin, and zinc finger E-box binding homeobox (ZEB) expression, while E-cadherin expression was downregulated (Fig. 8g, i). Conversely, the overexpression of PDGFD, in either non-metastatic osteosarcoma or metastatic osteosarcoma, resulted in a decrease in MMP9, β-catenin, and ZEB expression, while E-cadherin expression was increased (Fig. 8h, j). These findings suggest that PDGFD may play a role in regulating the metastatic potential of osteosarcoma through the EMT pathway.

## Discussion

Our finding provides a novel and comprehensive understanding for metastasis in osteosarcoma. We found that osteosarcoma samples consist of osteoblasts, osteocytes, osteoclasts, fibroblasts, pericytes, endothelial cells, myeloid cells, T cells, B cells and proliferating cells, but there was a great deal of variability between the different samples in the proportions of the cellular composition. Intercellular communication analysis suggested that the PDGF signaling pathway was present in the non-metastatic osteosarcoma primary sample. In contrast, no significant PDGF signaling was seen in metastatic osteosarcoma primary lesion sample. And the results were corroborated by the GSE152048 dataset, confirming the prominence of PDGFD-PDGFRB as a common ligand-receptor pair.

PDGFD is the novel member of PDGF family discovered in 2001 [40]. It triggers the activision of downstream signaling pathways via binding and activating PDGFRB [41, 42]. PDGFD was found to be associated with tumor development in a variety of malignancies of epithelial origin, such as cholangiocarcinoma [43], prostate cancer [44, 45], breast cancer [46, 47], ovarian cancer [48], colon cancer [49], laryngeal cancer [50], renal cancer [51], and so on. However, conflicting results were observed in other studies regarding the impact of PDGFD in metastasis. The deletion or knockdown of PDGFD gene impedes the differentiation of embryonic stem cells into endothelial cell lineage while promoting their self-renewal. Conversely, the overexpression of PDGFD gene triggers the differentiation of embryonic stem cells towards endothelial cells [52]. The capacity of PDGFD to enhance the expression of tissue inhibitor of metalloprotease 1 (TIMP-1) leads to the reduction of gelatinase activity of MMP2 and MMP9, as observed through gelatinase zymography [53]. PDGFD exhibits the ability to elevate interstitial fluid pressure, facilitate macrophage recruitment, and promote blood vessel maturation during angiogenesis [54], and overexpression of PDGFD resulted in an increase in perivascular cell coverage and the normalization of tumor blood vessels, thereby facilitating the penetration of doxorubicin into the tissue and enhancing its efficacy as a treatment [47]. PDGFD has the ability to induce the expression of IFN-γ, TNF-α, perforin, and CD107a, and also mediate the survival of human NK cells in response to interleukin-15 [55]. Additionally, a study examining the expression levels of PDGF ligands and receptors in 255 patients with soft tissue sarcoma discovered that high levels of PDGFD were associated with a decreased risk of metastasis in both univariate and multivariate analyses [56].

Little is known about PDGFD in osteosarcoma other than it had predictive power for the chemotherapy response [39]. To verified the findings based on scRNA-seq data, IHC was used to detect PDGFD and PDGFRB expression in non-metastatic and metastatic osteosarcoma primary lesions, and found that PDGFD was mainly expressed in osteosarcoma cells, while the PDGFRB expression was observed in both osteosarcoma cells and stromal cells. The distribution of PDGFD and PDGFRB in osteosarcoma were consistent with their distribution in Rhabdomyosarcoma as described before [57]. The positive rate of PDGFD was significantly higher in non-metastatic osteosarcoma primary lesions than that in metastatic primary lesions, and the founding agreed with the conclusion of Brahmi M et al., the relationship between high levels of PDGFD and a reduced risk of metastasis in the study of soft tissue sarcoma [56]. As a next step, multi-color immunofluorescence staining of PDGFD, PDGFRB, and COL1a1 was performed to identify the relationship of location, and co-localization of PDGFD and PDGFRB is indicated, suggesting that PDGFD is binding to PDGFRB. Further comparative analysis of the expression of PDGFD and PDGFRB with clinical characteristics indicated no significant correlation between either of them and gender, age, tumor site, pathological staging, neoadjuvant chemotherapy, maximum tumor diameter, necrosis rate, local recurrence, or distant metastasis. Survival curves demonstrated that the high level PDGFD group had significantly longer metastasis-free survival time and overall survival time compared to the low-level PDGFD group. Conversely, the expression level of PDGFRB did not show a significant correlation with metastasis-free survival time and overall survival time, implying that PDGFD may serve as a prognostic factor for MFS and OS.

Matrix metalloproteinases (MMPs) are enzymes that degrade various protein components of the extracellular matrix, thereby disrupting the histological barrier of cell invasion. These enzymes play a critical role in tumor cell invasion, angiogenesis, and metastasis [58]. Among the MMP family, MMP9 is an important member. PDGFD was observed to reduce the mRNA level of MMP9, as confirmed by RT-qPCR assay [53], which is consistent with the RT-qPCR results obtained in the present study. Additionally, gelatin zymography assay demonstrated that PDGFD also decreased the activity of MMP-9 [53]. It is worth noting that MMPs have also been implicated in the process of epithelial-mesenchymal transition (EMT), which is a hallmark of cancer progression to metastasis [59, 60]. E-cadherin, as an epithelial marker, is a key molecule in EMT. Several studies have confirmed that inhibition of E-cadherin promotes metastasis in osteosarcoma [61–64]. Cadherin-catenin complexes are integral components of the adherens junctions crucial for cell-cell adhesion [65]. Tyrosine phosphorylation of β-catenin induced it dissociated from the complexes, followed by loss of anchor attachment in E-cadherin and β-catenin, leading to increased motility and invasiveness [66]. ZEB plays a crucial role in the initiation of EMT. ZEB family members (ZEB1 and ZEB2) promote EMT by suppressing expression of E-cadherin, and overexpression of them were intimately associated with the genesis and metastasis of tumors [67]. t has been shown that ZEB2 is overexpressed in osteosarcoma [68]. Furthermore, A high expression of ZEB1 is highly correlated with invasion and metastasis of osteosarcoma [69]. In this study, Manipulating PDGFD expression demonstrated altered invasive abilities. Furthermore, the downregulation of PDGFD led to a significant upregulation of MMP9, β-catenin, and ZEB expression, while E-cadherin expression was downregulated in both cell types. Conversely, the overexpression of PDGFD resulted in a decrease in MMP9, β-catenin, and ZEB expression, while E-cadherin expression was increased. These findings suggest that PDGFD may play a role in regulating the metastatic potential of osteosarcoma through the EMT pathway.

This study utilized scRNA-seq analysis to identify the presence of PDGFD in non-metastatic osteosarcoma. Additionally, organoids of both non-metastatic and metastatic osteosarcoma were constructed, and immunohistochemistry and multicolor immunofluorescence techniques were employed to validate the distributional characteristics, expression, and ligand-receptor binding of PDGFD and PDGFRB. Furthermore, transwell assays and RT-qPCR assays were conducted using osteosarcoma primary cells to confirm that PDGFD potentially inhibits osteosarcoma metastasis via the EMT pathway. However, this study provides preliminary evidence supporting the inhibitory effect of PDGFD on osteosarcoma metastasis. Nonetheless, the precise interaction between osteosarcoma and fibroblasts remains unclear, necessitating further investigation into the downstream pathway of PDGFD-PDGFRB. Additionally, the study would benefit from an expanded analysis of immunohistochemistry and clinical data to enhance its credibility. These efforts are crucial in order to deepen our understanding of the metastatic mechanism of osteosarcoma and establish a foundation for the treatment of metastatic osteosarcoma.

## Electronic supplementary material

Below is the link to the electronic supplementary material.

Supplementary Material 1

## Data Availability

scRNA-seq data have been uploaded to the Gene Expression Omnibus repository: https://www.ncbi.nlm.nih.gov/geo/query/acc.cgi?&acc=GSE250015.
