# Supplementary material for: Exploring the impact of PDGFD in osteosarcoma metastasis through single-cell sequencing analysis
Source: Cell Oncol (Dordr). 2024 Apr 23;47(5):1715–33. doi: 10.1007/s13402-024-00949-3 (PMC11467127; doi:10.1007/s13402-024-00949-3)
Supplement: Supplementary file 1 — Supplementary Material 1 [file 13402_2024_949_MOESM1_ESM.docx]

### Supplementary information


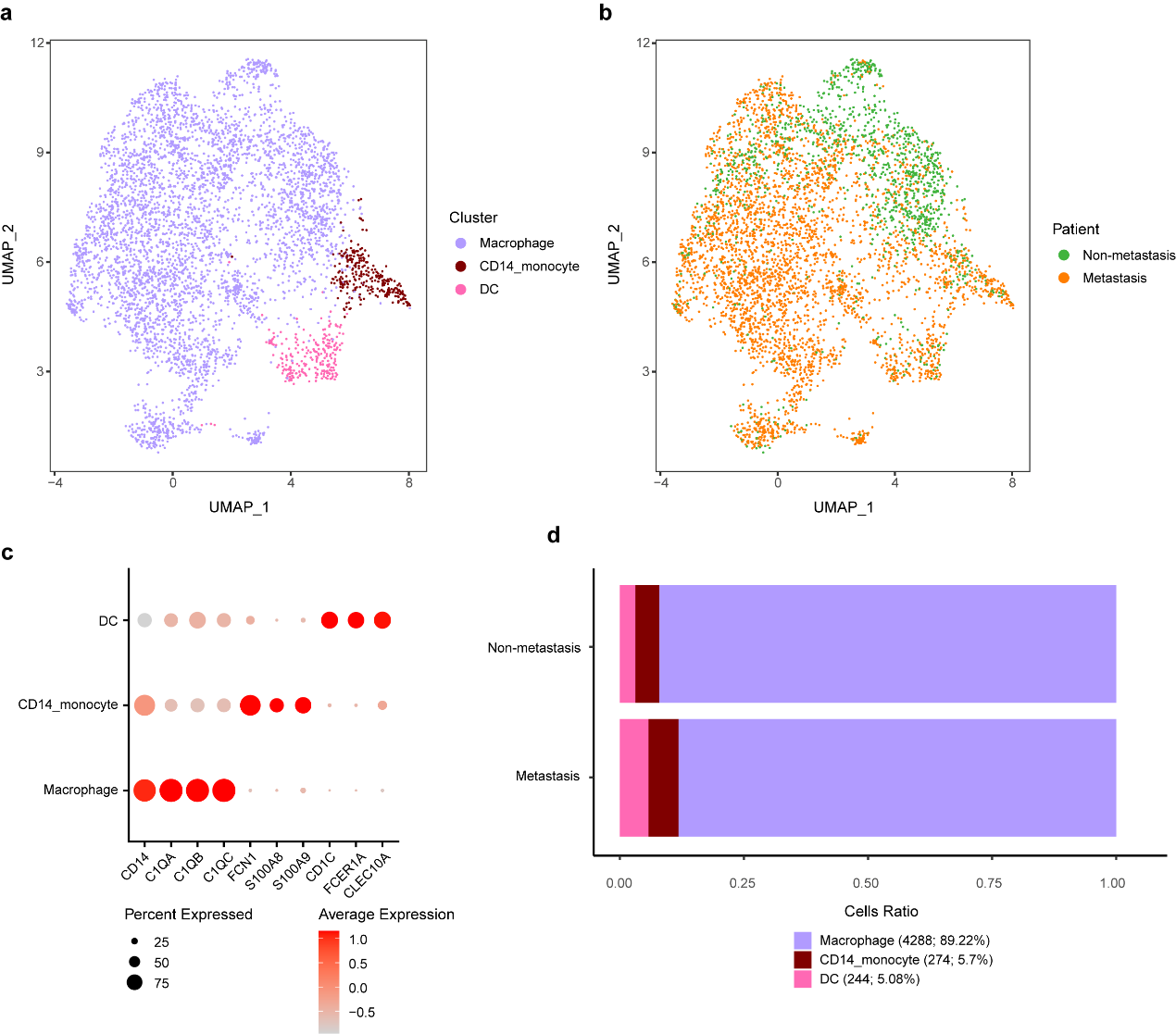


**Supplementary Fig. 1 Re-clustering of Myeloid cells.** **a** UMAP plot displayed the identified cells types. **b** UMAP of two different types of samples. **c** Dot plots showed the signature gene expressions across the cell types. **d** The relative proportions of all cell types in each sample.


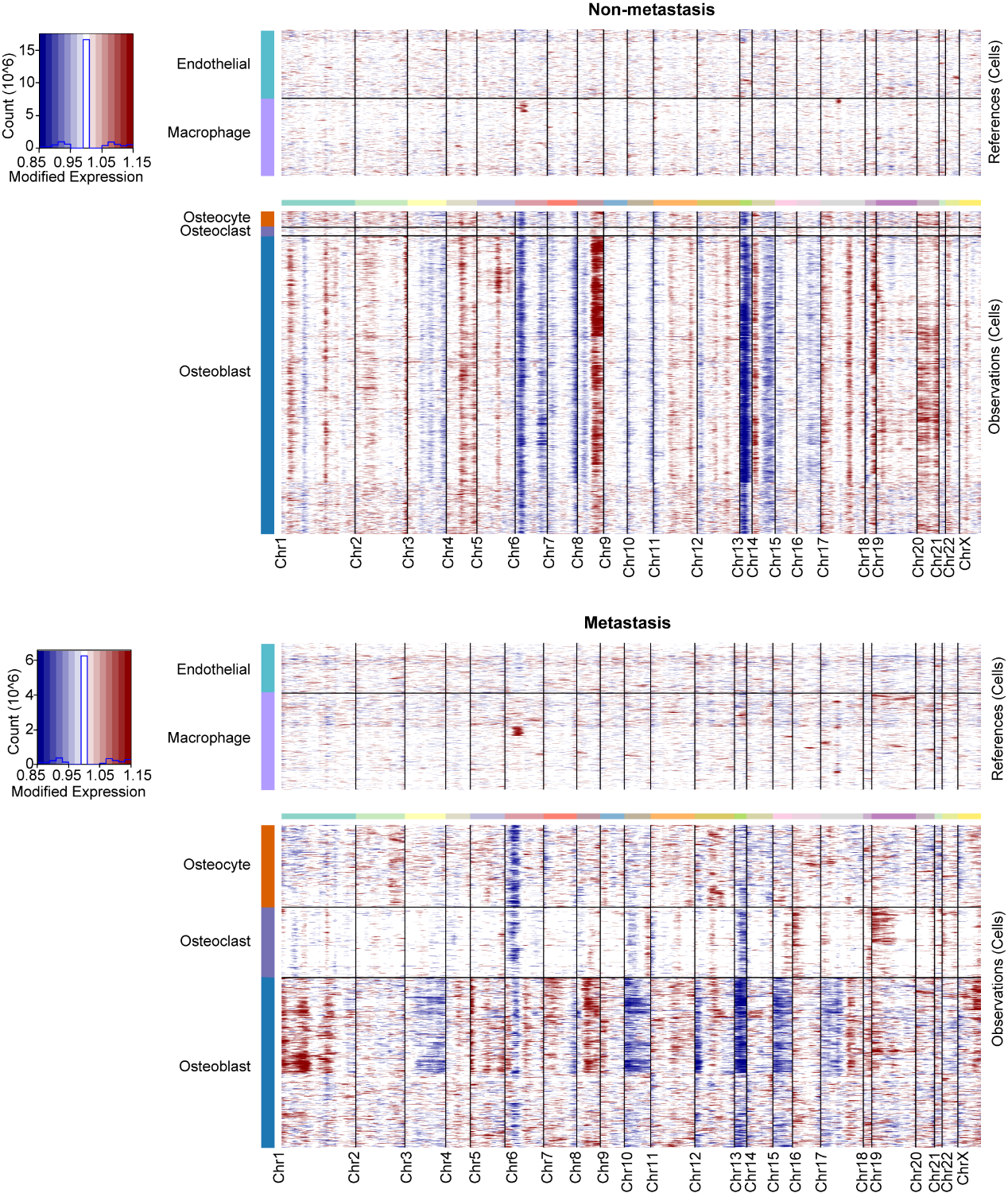


**Supplementary Fig. 2 CNV was present in osteoblasts, osteoclasts and osteoblasts.** The gene expression is colour-coded from 0.85 (blue) to 1.15 (red).


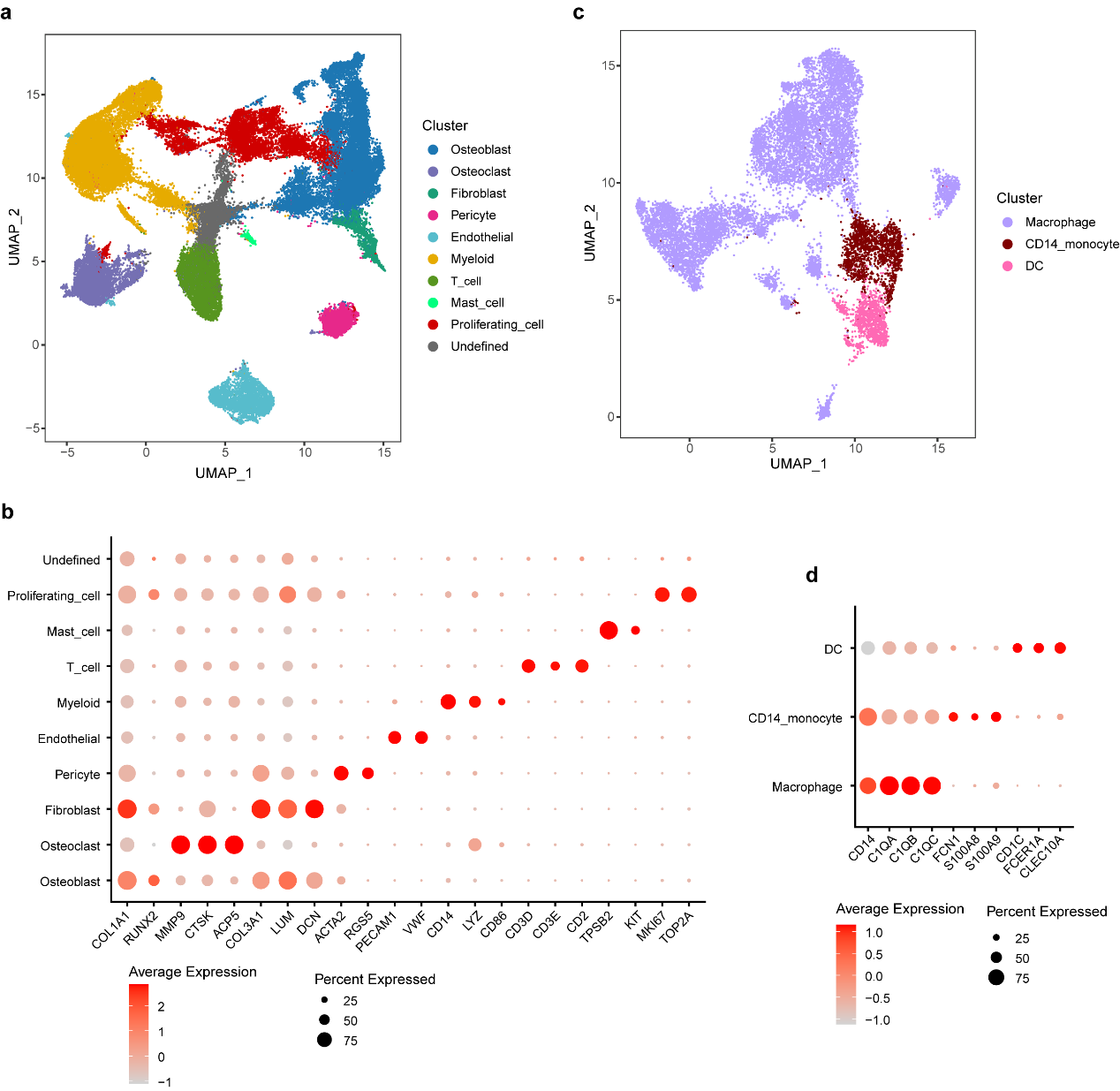


**Supplementary Fig. 3** **The identification of cells types in GSE 152048 dataset.** **a** UMAP plot displayed ten identified cells types. **b** Dot plots showed the signature gene expressions across the 10 cell types. **c** Subcluster diversity of Myeloid cells. **d** Dot plots showed the signature gene expressions across the 3 Myeloid cells types.


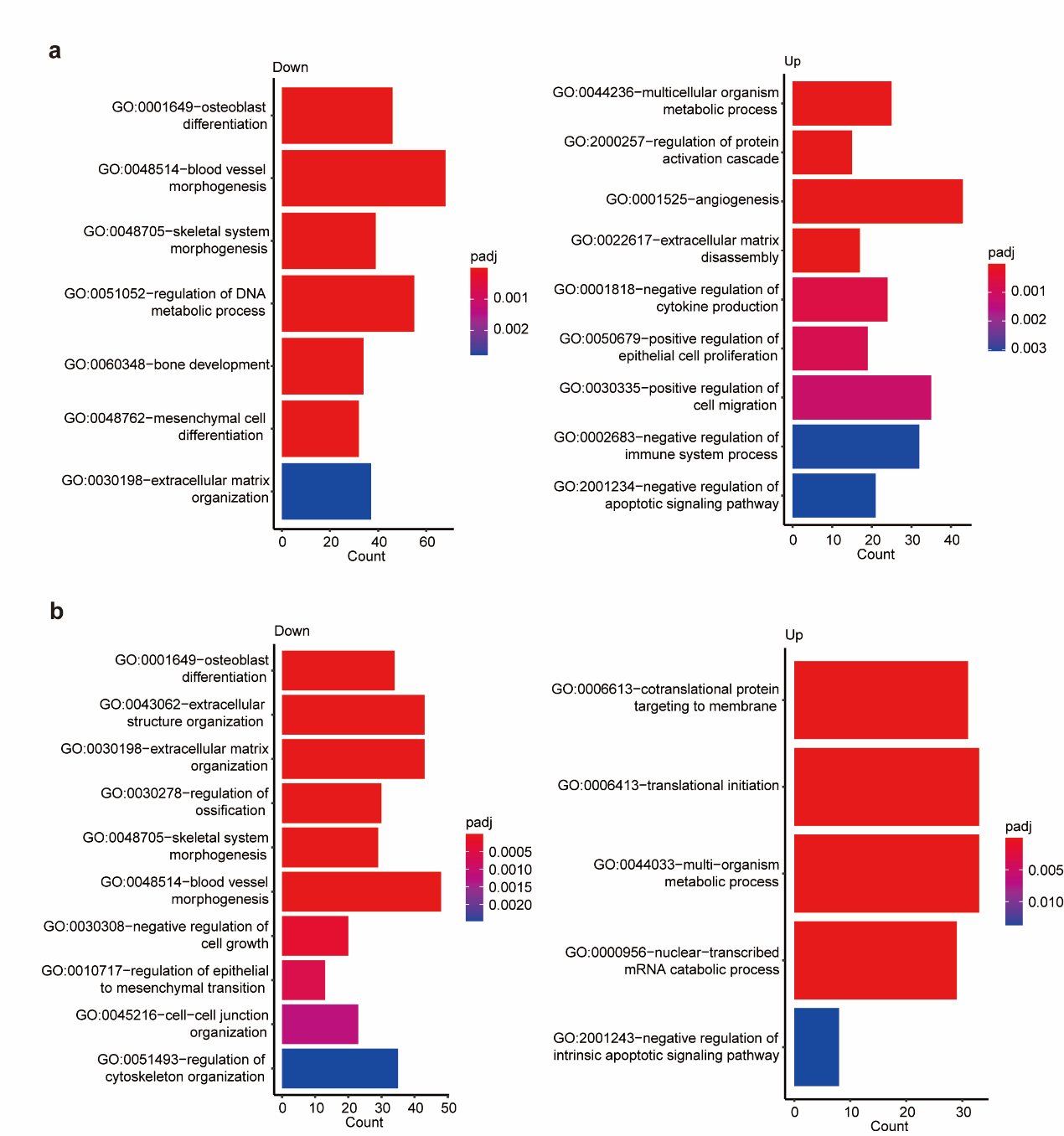


**Supplementary Fig. 4 Bar plot of GO analysis.** **a** Enriched GO BP terms in fibroblasts. **b** Enriched GO BP terms in osteoblasts.


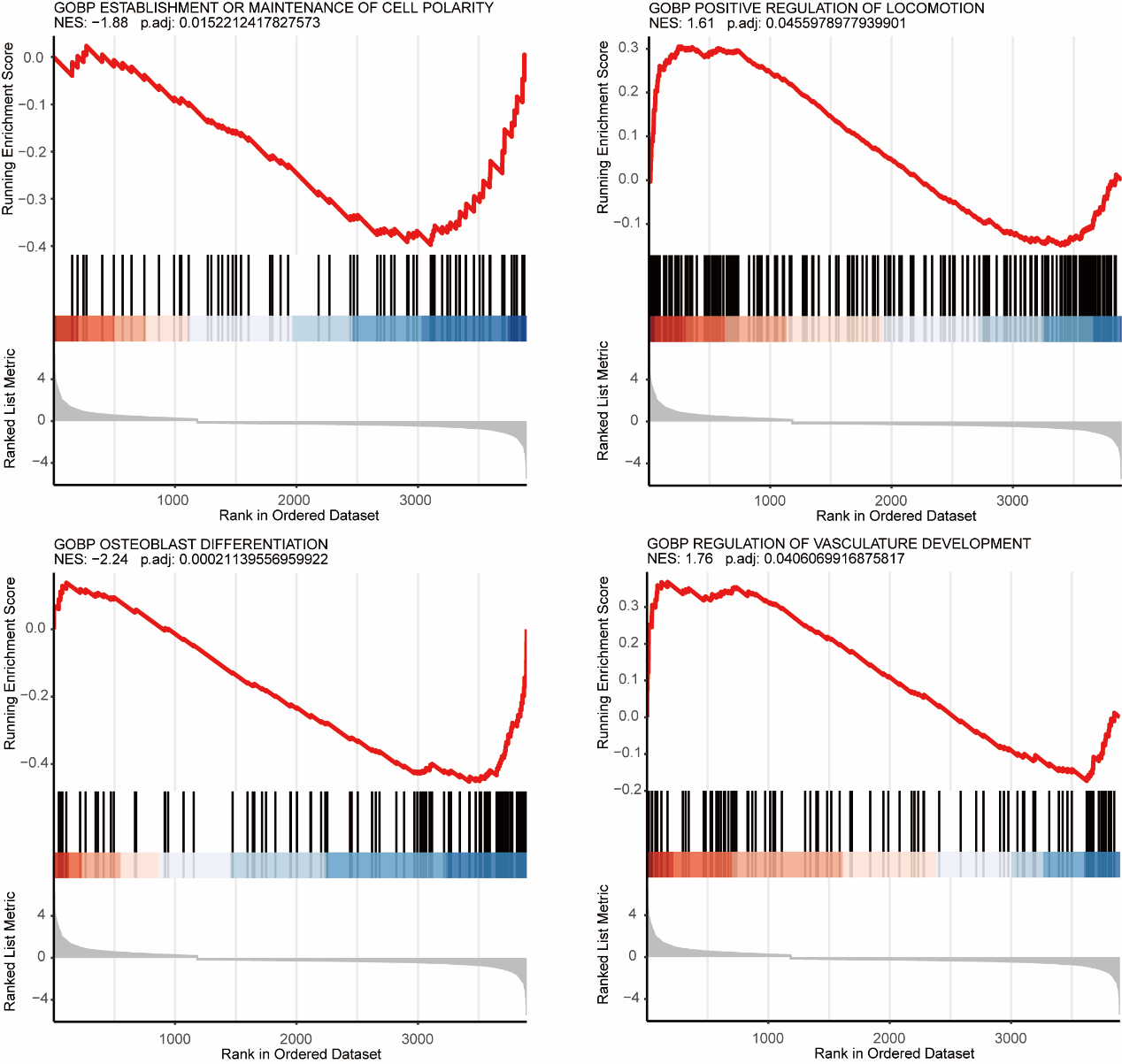


**Supplementary Fig. 5 Enrichment of GO BP terms by GSEA analysis in fibroblasts.**


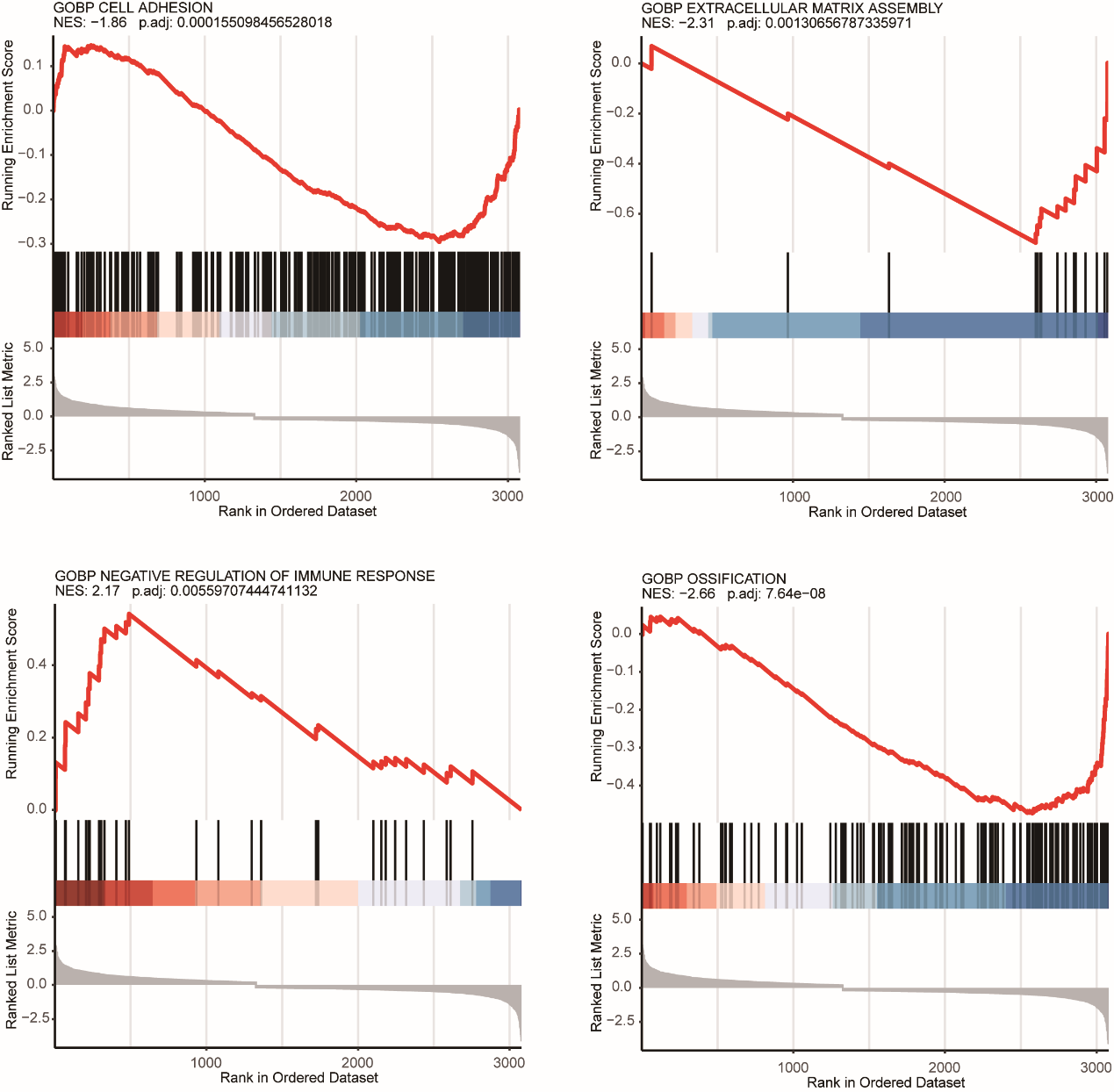


**Supplementary Fig. 6 Enrichment of GO BP terms by GSEA analysis in osteosarcoma.**

**
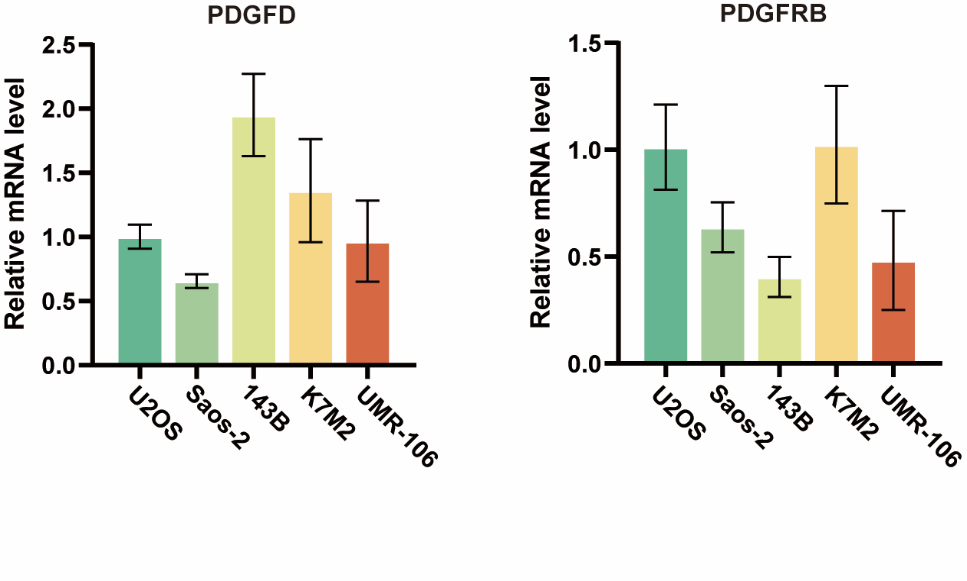
**

**Supplementary Fig. 7 The mRNA levels of PDGFD and PDGFRB in osteosarcoma cell lines.**

**Supplementary Table 1 Clinical characteristics of patients with osteosarcoma**

| ID | Gender | Age  (year) | Location | Pathology | Neoadjuvant chemotherapy | Diameter(cm) | Necrosis rate | Metastasis |
| --- | --- | --- | --- | --- | --- | --- | --- | --- |
| 1 | Female | 39 | Femur | Conventional | Yes | 13*7*5 | 40% | No |
| 2 | Male | 16 | Tibia | Conventional | Yes | 6*5*5 | 70% | Yes |
| 3 | Male | 8 | Femur | Conventional | Yes | 5*3*2 | 60% | No |
| 4 | Female | 9 | Radius | Conventional | Yes | 8*3*3 | 80% | No |
| 5 | Female | 13 | Tibia | Conventional | Yes | 5*3*2 | 50% | No |
| 6 | Male | 19 | Tibia | Conventional | Yes | 6*4*2 | 65% | No |
| 7 | Female | 18 | Femur | Conventional | Yes | 10*7*7 | 68% | No |
| 8 | Male | 13 | Femur | Conventional | Yes | 11*6*6 | 20% | Yes |
| 9 | Male | 30 | Femur | Conventional | Yes | 6*4*3 | 40% | Yes |
| 10 | Female | 19 | Femur | Conventional | Yes | 9*6*5 | 80% | Yes |
| 11 | Male | 18 | Humerus | Conventional | Yes | 17*7*6 | 80% | Yes |
| 12 | Female | 39 | Femur | Conventional | Yes | 8*5*3 | 50% | Yes |
| 13 | Male | 21 | Femur | Conventional | Yes | 10*8*3 | >90% | Yes |
| 14 | Female | 8 | Femur | Conventional | Yes | 5*3*3 | >90% | No |
| 15 | Female | 9 | Humerus | Conventional | Yes | 5*5*4 | 30% | No |
| 16 | Male | 14 | Humerus | Conventional | Yes | 10*6*6 | 10% | Yes |
| 17 | Female | 20 | Femur | Conventional | Yes | 9*7*5 | 30% | Yes |
| 18 | Female | 61 | Femur | Conventional | No | 7*7*6 | - | No |
| 19 | Male | 52 | Radius | Conventional | Yes | 8*5*5 | 27% | Yes |
| 20 | Male | 17 | Femur | Conventional | Yes | 17*5*3 | 80% | Yes |
| 21 | Male | 16 | Femur | Conventional | Yes | 12*7*7 | 90% | No |
| 22 | Male | 27 | Femur | Conventional | Yes | 15*5*4 | 90% | No |
| 23 | Female | 10 | Tibia | Conventional | Yes | 7*4*2 | >90% | No |
| 24 | Male | 14 | Humerus | Conventional | Yes | 11*7*3 | 30% | Yes |
| 25 | Male | 16 | Femur | Conventional | Yes | 11*7*5 | 80% | Yes |
| 26 | Male | 21 | Tibia | Conventional | Yes | 8*5*5 | 90% | No |

**Supplementary Table 2 The primer sequences for RT-qPCR reactions**

| Gene name | Forward (5’-3’) | Reverse (5’-3’) |
| --- | --- | --- |
| PDGFD (human) | TACAGTTGCACTCCCAGGAAT | CTTCCAGTTGACAGTTCCGCA |
| PDGFRB (human) | CCATCAGCAGCAAGGCGA | CCAGAAAAGCCACGTTGGTG |
| MMP9 (human) | TCTATGGTCCTCGCCCTGAA | TTGTATCCGGCAAACTGGCT |
| β-catenin (human) | TGGAACATGAGATGGGTGGC | GTGTTCTACACCATTACTCAATTCT |
| E-cadherin (human) | TGGGCCAGGAAATCACATCC | TGCAACGTCGTTACGAGTCA |
| ZEB (human) | GGATCAGGTCGTCGGTCTTG | TTACACCCAGACTGCGTCAC |
| GADPH (human) | TGTTCCTACCCCCAATGTGTC | TGAAGTCGCAGGAGACAACC |
| PDGFD (mouse) | GTTGACAGTTCCGCAACCAC | CCAACCTCAGGAGAGATGACT |
| PDGFRB (mouse) | TCAGGGGCAGATGGGACATA | ACCTCAAAAGTAGGTGTCCACG |
| GADPH (mouse) | CTCGTGGTTCACACCCATCA | GCCTCCTCCAATTCAACCCT |
| PDGFD (rat) | CTCCGGTACAAGTCTGTGAGG | ATCGGGACACTTTTGCGACT |
| PDGFRB (rat) | TCGCTGGTGGTCATAGGGTA | GTGTTCCGACTCCTCCTTCG |
| GADPH (rat) | ATTCGAGAGAAGGGAGGGCT | GCGAGATCCCGCTAACATCA |

**Supplementary Table 3 The cell composition of osteosarcoma samples**

| Cell type | Non-metastasis | Metastasis | Total |
| --- | --- | --- | --- |
| Osteoblast | 2971 | 889 | 3860 |
| Osteocyte | 159 | 430 | 589 |
| Osteoclast | 84 | 368 | 452 |
| Fibroblast | 1204 | 600 | 1804 |
| Pericyte | 401 | 1367 | 1768 |
| Endothelial cell | 1046 | 1617 | 2663 |
| Myeloid | 1280 | 3526 | 4806 |
| T cell | 185 | 839 | 1024 |
| B cell | 1 | 48 | 49 |
| Proliferation cell | 535 | 629 | 1164 |

**Supplementary Table 4 The cell composition of myeloid cells**

| Cell type | Non-metastasis | Metastasis | Total |
| --- | --- | --- | --- |
| Macrophage | 1178 | 3110 | 4288 |
| CD14^+^ monocyte | 62 | 212 | 274 |
| Dendritic cell | 40 | 204 | 244 |

**Supplementary Table 5 Number of cells’ interactions in non-metastatic osteosarcoma**

| Cell type | Endothelial cell | Fibroblast | Macrophage | Osteoblast | Osteoclast | Osteocyte | Pericyte | T cell |
| --- | --- | --- | --- | --- | --- | --- | --- | --- |
| Endothelial cell | 2 | 2 | 8 | 1 | 6 | 0 | 2 | 5 |
| Fibroblast | 8 | 13 | 9 | 10 | 9 | 3 | 7 | 4 |
| Macrophage | 3 | 2 | 8 | 1 | 8 | 0 | 2 | 5 |
| Osteoblast | 4 | 6 | 4 | 4 | 3 | 1 | 3 | 3 |
| Osteoclast | 1 | 2 | 4 | 1 | 3 | 0 | 2 | 3 |
| Osteocyte | 1 | 3 | 4 | 0 | 3 | 0 | 1 | 3 |
| Pericyte | 8 | 8 | 8 | 7 | 8 | 2 | 5 | 4 |
| T cell | 2 | 2 | 4 | 1 | 5 | 0 | 2 | 3 |

**Supplementary Table 6 Number of cells’ interactions in metastatic osteosarcoma**

| Cell type | Endothelial cell | Fibroblast | Macrophage | Osteoblast | Osteoclast | Osteocyte | Pericyte | T cell |
| --- | --- | --- | --- | --- | --- | --- | --- | --- |
| Endothelial cell | 5 | 5 | 13 | 1 | 14 | 2 | 3 | 5 |
| Fibroblast | 8 | 5 | 16 | 1 | 10 | 2 | 2 | 4 |
| Macrophage | 3 | 1 | 14 | 0 | 10 | 2 | 2 | 4 |
| Osteoblast | 1 | 1 | 5 | 0 | 6 | 1 | 1 | 3 |
| Osteoclast | 2 | 1 | 10 | 0 | 11 | 1 | 2 | 5 |
| Osteocyte | 5 | 4 | 10 | 1 | 8 | 2 | 3 | 2 |
| Pericyte | 7 | 7 | 10 | 2 | 10 | 3 | 5 | 4 |
| T cell | 3 | 1 | 6 | 0 | 6 | 0 | 0 | 1 |
